# Influence of socio-demographic factors on medicinal plant knowledge among three selected ethnic groups in south-central Ethiopia

**DOI:** 10.1186/s13002-024-00672-1

**Published:** 2024-02-28

**Authors:** Sintayehu Tamene, Mesele Negash, Fortunatus Bulabo Makonda, Linley Chiwona-Karltun

**Affiliations:** 1 Wondo Genet College of Forestry and Natural Resources, Hawassa University, PO Box 05, Hawassa, Ethiopia; 2 College of Forestry, Wildlife, and Tourism, Sokoine University of Agriculture, Morogoro, Tanzania; 3 Department of Urban and Rural Development, Swedish University of Agricultural Sciences, Uppsala, Sweden; 4 Hawassa University, Hawassa, Ethiopia

**Keywords:** Ethiopia, Indigenous knowledge, Rural–urban interface, Socio-demographic variables, Traditional knowledge

## Abstract

**Background:**

The influence of socio-demographic variables was widely explored to evaluate their impact on indigenous and local ethnobotanical knowledge. However, the studies conducted in Ethiopia mainly focused on rural areas. They were limited to exploring and documenting ethnobotanical knowledge and the associated impacts of socio-demographic variables in rural–urban interface areas among ethnic groups. Hence, this study aimed to document plant-based indigenous and local ethnomedicinal knowledge and the associated impacts of socio-demographic variables among selected three ethnic groups in south-central Ethiopia.

**Methods:**

Ethnobotanical data were collected using semi-structured interviews with 189 key informants, floristic species inventories, and field observations. Quantitative approaches were used to evaluate the use values (UV) of the most important medicinal plants, the informant consensus factor (ICF), fidelity level (FL), relative popularity level (RPL), and rank-order priority (ROP). Statistical tests were applied to evaluate the influences of socio-demographic factors and associations between variables on local ethnobotanical knowledge across ethnic groups in different informant categories.

**Results:**

Statistical analysis revealed significant differences (p < 0.05) in the mean number of medicinal plants reported among age categories. There was also a positive association between the respondent's age and plant knowledge acquisition. *Croton macrostachyus* Hochst. ex Delile, *Albizia gummifera* C.A.Sm., *Zingiber officinale* Roscoe, *Aloe macrocarpa* Tod., *Gymnanthemum amygdalinum* (Delile) Sch.Bip., *Calpurnia aurea* (Aiton) Benth, and *Allium sativum* L. had the highest use values among ethnic groups. The highest informant consensus factor values were recorded for circulatory system disorders (0.68) followed by febrile illness and reproductive organ complications (0.66 each) across the three studied ethnic groups. The highest FL, RPL, and ROP values were noted for *Lactuca inermis* Forssk., *Moringa stenopetala* (Baker f.) Cufod., *Withania somnifera* (L.) Dunal*, Allium sativum* L., *Citrus limon* (L.) Osbeck*, Ricinus communis* L., *Schinus molle* L., *Antiaris toxicaria* (J.F.Gmel.) Lesch., *Brucea antidysenterica* J.F.Mill., *Echinops kebericho* Mesfin, *Ocimum jamesii* Sebald, *Afrocarpus falcatus* (Thunb.) C.N.Page, *Searsia natalensis* (Bernh. ex Krauss) F.A.Barkley, and *Ricinus communis* L. across ethnic groups in the study areas, which showed the conformity of knowledge on species curing potential and their prevalent uses.

**Conclusion:**

The study revealed that the ethnic groups of Gedeo, Oromo, and Sidama have considerable indigenous and local ethnobotanical knowledge practices. Statistical analysis shown high variation in the acquisition of local ethnobotanical knowledge among age groups, which boosted our understanding of the effects of socio-demographic factors on the local ethnobotanical knowledge dynamics. Thus, this finding advocates for efforts to repair the observed generation gap via continued professional support and educating local communities to preserve traditional knowledge and practices through systematic documentation.

## Introduction

Long before were scientific inquiry established, humans created, disseminated, and utilized information about the natural world [1]. Over millennia, indigenous peoples across the globe have developed, maintained, and evolved knowledge systems through direct interaction with biophysical and biological processes and species [1, 2]. As a result, knowledge held by people about their environment evolved gradually and accumulated throughout their histories [3]. Across the globe, including Ethiopia, this local knowledge was significantly dependent on generations’ constant connection with their surroundings and elders [3, 4] and regarded as a body of place-based knowledge accumulated and transmitted across generations within specific cultural contexts [1, 3, 5]. According to [5–7], indigenous and local knowledge is not only direct observation and contact with the environment but also a wide range of cultural and spiritual knowledge and values that enhance human–environment relationships. Ethnobotanical studies conducted elsewhere explained the associations between socio-demographic factors and knowledge of plant use [4, 8–12]. Some of them revealed that age and ethnobotanical knowledge have a direct relationship and that ethnobotanical knowledge accumulation increases with an individual’s age [8–10, 13] and is widely used among communities with poor health facilities [12]. Others explained the influences of gender and education level: males and lower-grade attendees were more familiar with the medicinal values of local flora [4, 8], and traditional healers possess richer ethnobotanical knowledge than laypeople [10, 13]. Thus, the cultural variables seem essential in explaining and determining plant use knowledge [5, 10, 14].

Ethiopia is one of the world’s most ethnically and culturally diverse country, with over 70 different languages spoken across and more than 80 distinct ethnicities [16, 17]. Several ethnobotanical studies have been conducted to document traditional medicinal plant knowledge and the associated factors elsewhere in the country [4, 8–10, 13, 16, 19–25]. However, they are insignificant when compared to the 80 diverse ethnolinguistic communities, and most of them are largely unexplored and limited to rural areas. According to CSA [25], south-central Ethiopia is home to diverse ethnic groups, representing more than half of the country's indigenous ethnic communities; but, evidences from [8, 18–20, 23, 24, 26–29] reveal that studies conducted on medicinal plants have so far focused in the south and southwestern parts, covering only a few out of the estimated 45 or more socio-cultural (language) groups. Thus, medicinal plant resources and indigenous knowledge about the use of medicinal plants in south-central Ethiopia, particularly in the current study's peri-urban area, are inadequate.

As a result, the current study was conducted to fill this gap by documenting the abundance of indigenous and local ethnobotanical knowledge and understanding the corresponding socio-demographic drivers among the three ethnic groups in south-central Ethiopia. Specifically, the study aimed at (i) documenting plant-based indigenous and local ethnomedicinal knowledge of the Gedeo, Oromo, and Sidama ethnic groups against human ailments in Dilla, Shashemene, and Hawassa peri-urban areas, respectively; (ii) evaluating the impact of socio-demographic variables on medicinal plant knowledge among the three studied ethnic groups; (iii) determining the use values (UV) of the most important medicinal plants among the three studied ethnic groups; and (iv) identifying potential medicinal plant species among the three ethnic groups for future phytochemical and pharmacological investigations. The hypothesis is that medicinal plant knowledge varies depending on the socio-demographic variables among ethnic groups. The study will contribute scientific information about the medicinal flora and associated ethnobotanical knowledge, as well as understanding the influences of socio-demographic variables on local and indigenous medicinal plant knowledge in the rural–urban interface areas of south-central Ethiopia.

## Materials and methods

### Description of the study area

**Fig. 1:**
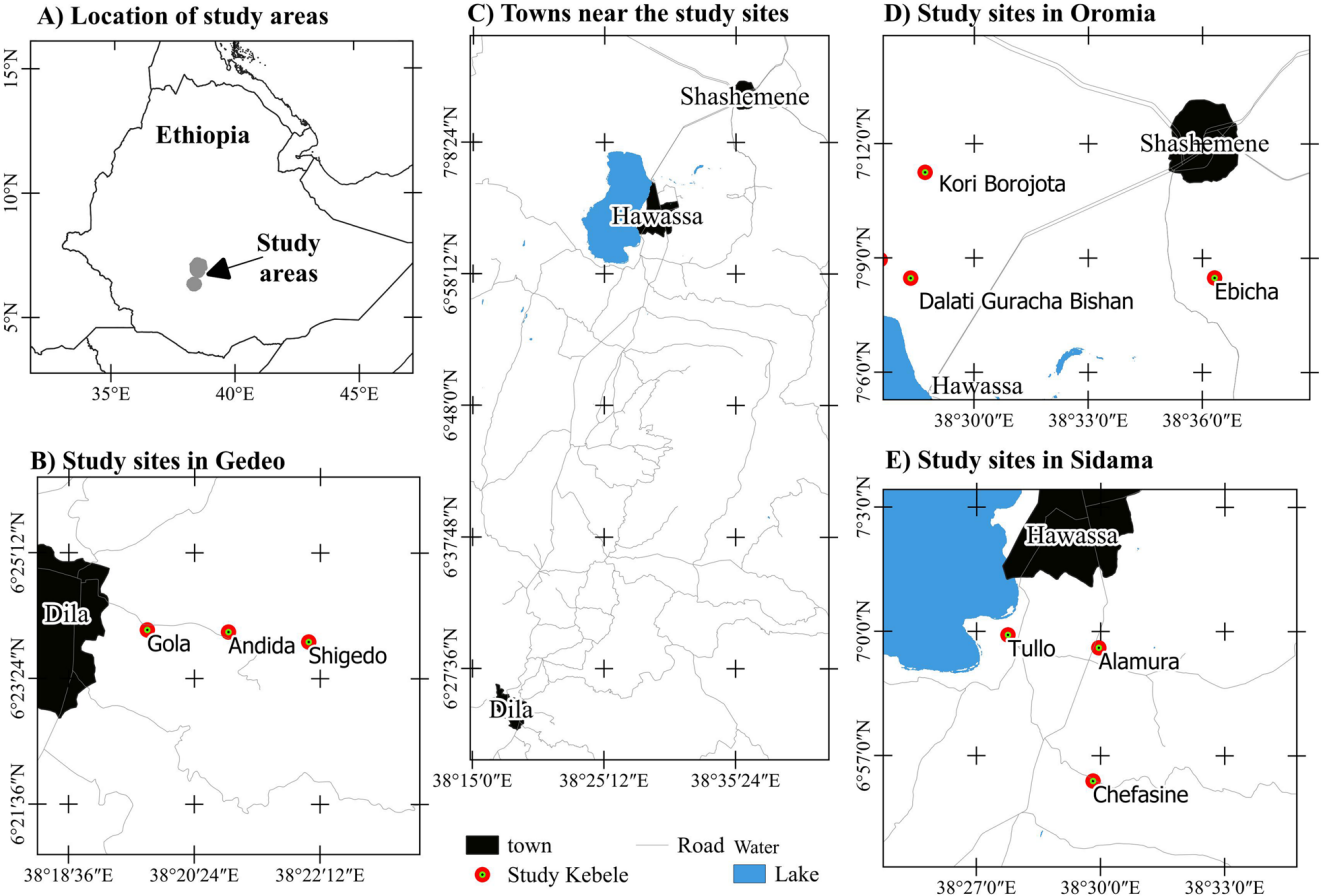
Map of the study sites Dilla, Shashemene, and Hawassa peri-urban areas

The study was conducted in three selected sites of neighboring ethnic groups in the south-central part of Ethiopia: Hawassa, Shashemene, and Dilla. Administratively, Hawassa district is part of the Sidama National Regional State, whereas Shashemene and Dilla districts are parts of the Oromia and Southern National Regional States, respectively (Fig. 1). Nine peri-urban kebeles (lowest administrative units; three from each study site) were purposely selected at different distances from the peri-urban administrative parts of Hawassa, Shashemene, and Dilla (Fig. 1). These areas were chosen because the ethnic groups living in the districts have coexisted and interacted for many years in specific regions of south-central Ethiopia. Additionally, they are geographically close to the town, have similar urbanization pressures, and are facing aggressive degradation of natural resources owing to urbanization [30–33]

Hawassa is situated 273 km south of Addis Ababa [34], at 6°55′–07°06′N and 38°25′–38°33′E, with elevation ranges of 1656 to 2137 m a.s.l.[35]. The borders of Hawassa area are defined by Lake Hawassa to the west, Oromia National Regional State to the north, Wondo Genet and Malga districts to the east, and Shebedino and Gorge districts to the south. Hawassa had 15,720 hectares of land within its administrative boundary, while only 6,465 hectares (24.4%) were demarcated within the municipal boundary and planned as urban land, while the rest is rural land [34]. Administratively, the city was organized into three tiers of administration: which divided into 8 sub-cities and 32 kebeles (lowest administrative units) (Hawassa City Administration annual unpublished report, 2019). Among the sub-cities, Hawella-Tulla and its 12 kebeles were categorized as rural and rural–urban interface areas, where the current study was conducted (Fig. 1). Residents of the study area are ethnically and religiously diverse. The majority of indigenous and local people living in the area are Sidama (48.68%), followed by Amhara (15.43%), Welaita (13.9%), Oromo (5.21%), Gurage (4.33%) and others (12.45%), CSA [25]. The major language spoken in the area belongs to the Sidama ethnic group ‘*Sidamu afoo’* (47.97%), followed by Amharic (31.01%), Welaita (9.58%), Afan Oromo (2.53%), Gurage (1.98%), Kembata (1.82%), and others (5.09%). More than half of the people in the research area practice the Protestant religion (52.71%), followed by Ethiopian Orthodox Christianity (39.99%), Islam (7.3%), and Catholicism (3.78%). According to the CSA [25] population forecasts, the projected population for 2022 was 555,480, of whom 277,032 were males and 278,448 were females.

Shashemene district is located at 7°04′50″ to 7°22′45″N and 38°23′00″ to 38°48′00″E. Which is 250 km to the south of Ethiopia's capital city, Addis Ababa, and 25 km north of Hawassa, the capital city of Sidama National Regional State [36]. Hawassa city borders it to the south, Seraro to the west, Arsi Negele to the north, and Arsi Zone to the east. Its elevation ranges from 1,500 to 2,300 m a.s.l. [36]. The district had a 767.9km^2^ area with 458.3/km^2^ population density [25]. The district rural and rural–urban interface areas have assembled into 28 kebeles (lowest administrative units), where the current study was conducted (Fig. 1). Residents of the study area were ethnically and religiously diverse. The Oromo ethnic group makes up the majority of the indigenous inhabitants in the district (74.11%), followed by the Amhara (9.26%), Welaita (95%), Kembata (2.3%), Gurage (2.13%), and others accounted for 7.2%, CSA [37]. The major language spoken in the area belongs to the Oromo ethnic group ‘*Afan Oromo’* (71.7%), followed by Amharic (18.23%), Welaita (3.49%), Kembata (1.52%), Gurage (1.14%), and others (2.02%). The majority of the inhabitants were Islamic religion followers, with 69.38% of the population, followed by Ethiopian Orthodox Christianity (23.5%), Protestantism (5.62%), and Catholicism (1.05%). According to the CSA [25] population forecasts, the projected population for 2022 was 351,898, of whom 174,711 were males and 177,187 were females in the district (Table 1).

**Table 1:** Detailed locations of the study sites

Study sites	Longitude (o)	Latitude (o)	Elevation	Agro-ecology
Kori Borojota	38.480479305928	7.187481904610	1806	Moist mid-highland (Moist Weina Dega)
Dalati Guracha Bishan	38.476134272769	7.142100771683	1744	Moist mid-highland (Moist Weina Dega)
Ebicha	38.602659011844	7.136859913447	2043	Moist mid-highland (Moist Weina Dega)
Tullo	38.459627749373	6.996525786840	1700	Moist mid-highland (Moist Weina Dega)
Alamura	38.497456580871	6.989165780928	1704	Moist mid-highland (Moist Weina Dega)
Chefasine	38.495170076222	6.939687380283	1926	Moist mid-highland (Moist Weina Dega)
Gola	38.327582906735	6.401188224290	1760	Wet mid-highland (Wet Weina Dega)
Andida	38.349498162680	6.400104445130	1883	Wet mid-highland (Wet Weina Dega)
Shigedo	38.365856530154	6.398218606985	1981	Wet mid-highland (Wet Weina Dega)

The agro-ecologies are described based on the agro-climatic zone classification of Ethiopia, Tesemma [39]

Dilla district was located in southern Ethiopia, 359 km from the capital city, Addis Ababa [38], and an altitude range of 1,350 to 2,550 m a.s.l. It is situated at 6°15′05" to 6°26′35 N and 38°15′55" to 38°24′02"E. The district had a 122.3 km^2^ area with a 1,047/km^2^ population density [25]. The district rural and rural–urban interface areas were assembled into 19 kebeles (lowest administrative units), where the current study was carried out (Fig. 1). The district residents are heterogeneous, both in ethnicity and in religion. The majority of indigenous people inhabiting the area belong to the Gedeo ethnic group (73.5%), followed by the Amhara (6.98%), Oromo (6.37%), Sidama (3.34%), Silte (2.33%), and others (7.48%), CSA [37]. The Gedeo ethnic group language '*Gedeoffa'* was spoken widely (73.22%), followed by Amharic (13.5%), Afan Oromo (5.43%), Sidamu afoo (3.25%), Silte (1.39%), and others (3.21%). The majority of the people were Protestants religion followers, accounting for 83.13% of the population, followed by traditional belief (7.81%), Ethiopian Orthodox Christianity (5.31%), Catholicism (1.16%), Islam (1.02%), and others (1.57%), CSA [25]. According to the CSA [25] population forecasts, the projected population for 2022 was expected to reach 128,050, of whom 64,276 were males and 63,774 were females in the district.

### Informant selection

To ensure a detailed representation of indigenous and local knowledge dynamics and plant uses, traditional healers of the three ethnic groups were selected based on their gender, age, experience, level of education, and religion. For the survey, 189 key informants (133 males and 56 females) with the age range of 35 to 77 were selected using purposive and snowball sampling techniques based on recommendations from local communities, local government heads, and development workers following [16, 17]. Of the total, 63 were from the Sidama (43 males and 20 females), 63 were from the Oromo (41 males and 22 females), and 63 were from the Gedeo ethnic group (49 males and 14 females). Informed consent has been obtained from all informants who served as informants before the start of the interviews.

### Ethnobotanical data collection and specimen identification

Ethnobotanical data were collected from January to May 2023, using semi-structured interviews with local traditional healers and inventories of plant species following an approach of [40–42]. The first session included information regarding the socio-demographic characteristics of the informants. Secondly, information related to the detailed ethnobotanical application of the local flora. Most of the interviews were carried out in local languages (Sidamu Afoo, Afan Oromo, and Gedeoffa) with the help of local translators or a language native to the respective research locations. Each ethnic group studied was an indigenous and local resident of the study area. All floristic voucher specimens were collected with the help of traditional healers and development professionals. Specimens were identified in the field and later confirmed at the National Herbarium of Addis Ababa University and Wondo Genet College of Forestry and Natural Resources, Hawassa University using taxonomic keys and flora [43–48]. The verified specimens in the National Herbarium were further checked using Plants of the World Online (https://powo.science.kew.org.) websites to confirm the correctness of the scientific names and author citations. Finally, the plants were dried, pressed, mounted on a herbarium sheet, and placed at Hawassa University's Wondo Genet College of Forestry and Natural Resources Herbarium.

### Data analysis

Both qualitative and quantitative data were analyzed using the ethnobotanyR package, Version 0.1.8, 2022. Age, gender, education, and religion were used to determine the impacts of socio-demographic variables on ethnobotanical knowledge. The Kruskal–Wallis chi-squared test, a non-parametric approach to the one-way ANOVA, was performed between age, education, religion, and the number of medicinal plants cited, and the Wilcoxon test for gender. The mean and standard deviation of the number of medicinal plants reported concerning the socio-demographic variables were evaluated. Regression analysis was conducted to determine the association between respondent's ethnobotanical knowledge and age. Quantitative ethnobotanical tools such as the informant consensus factor (ICF), use values (UV), fidelity level (FLs), relative popularity level (RPL), and rank-order priority (ROP) were also used for data analysis.

### Use Value index (UVi)

The use value index (UVI) was calculated to find out the relative importance of medicinal plant species following [49].

$$UV_{s} = \sum\limits_{{i = i_{1} }}^{{^{i} N}} {\sum\limits_{{u = u_{1} }}^{{^{u} NC}} {UR_{ui/N} } }$$

where ’Ui’ is the number of different uses mentioned by each informant i and ‘N’ is the total number of informants interviewed for the given plant species.

### Informant consensus factor (ICF)

The informant consensus factor was calculated to investigate the degree of homogeneity among informants for the plants to be used in each ailment category [50]. The ICF values vary from zero to one, with a high ICF achieved when one or a few plant species were reported to treat a certain condition by a large proportion of informants.

$$F_{{{\text{ic}}}} = \frac{{n_{{{\text{ur}}}} - n_{{\text{t}}} }}{{n_{{{\text{ur}}}} - 1}}$$

where ‘Nur’ is the number of use reports in each ailment category and ‘Nt’ is the total number of taxa used in each ailment category by all the informants.

The result of this factor ranges from 0 to 1. A high value (close to 1) indicates that relatively few plant species are used by a large proportion of people, and a low value indicates that the informants disagree on the plant species used to treat a category of illness.

### Fidelity level (FL)

The fidelity level is applied to determine which species are most frequently employed by the key informants to treat particular conditions. Higher FL values would indicate medicinal plants are more commonly used by the local communities, reveal the proportion of informants who reported using a specific plant species for the same purposes, and highlight the significance of the species for specific conditions. Following [51] and [52], all reported illnesses were arranged into major categories before determining the values using the formula [51].

$$FL_{s} = \frac{{N_{s} *100}}{{FC_{s} }}$$

where Ns is the number of informants that use a particular plant for a specific purpose and FCs is the total number of uses for the species.

### Relative popularity level (RPL)

The relative popularity level (RPL) is a ratio of the number of important use reports mentioning a certain plant species to the number of interviewees mentioning that taxon in any use reports. RPL values vary from zero to one, with one representing total popularity and zero representing unpopularity [53].

### Rank-order priority (ROP)

Relative popularity level (RPL) value multiplied by fidelity level (FL) value yielded the rank-order priority or accurate value of fidelity level (FL) (ROP = RPL * FL) [51]. A high ROP value suggests that the plant has great potential. It might be beneficial for screening plants for bioactive compounds.

## Results

### Socio-demographic characteristics and knowledge of traditional healers

According to interviews with key informants (Table 2), 189 medicinal plant species were collected and documented (Table 3). Among the 189 informants, the majority of participants were farmers, 155 (82%); others were merchants, 23 (12%); and students, 11 (6%). Male participants were outnumbered female participants (Table 2). About 70% of participants in this study were between the ages of 45 and 65. In comparison, those under 45 (young) and over 65 (elderly) accounted for 30% of the remaining population (Table 2). Illiterate and lower-grade informants were higher compared to high school attendees. Most interviewees were protestant religion followers, followed by Muslims and Orthodox Christians (Table 2).

**Table 2:** Demographic details of the participants from peri-urban areas of Dilla, Hawassa, and Shashemene

Socio-demographic features	Categories	Frequency	Percentage
Ethnicity	Gedeo	63	33.33
	Oromo	63	33.33
	Sidama	63	33.33
Age	35–44	33	17
	45–54	45	24
	55–64	88	47
	65 +	23	12
Gender	Male	133	70
	Female	56	30
Education	Illiterate	89	47
	Primary (1–8 grade)	81	43
	Secondary level	19	10
Religion	Protestant	101	53
	Orthodox	13	7
	Islam	75	40

**Table 3:** Medicinal plants mentioned by the three studied ethnic groups (Sidama (S), Gedeo (G), and Oromo (O)) in south-central Ethiopia (N = 189)

Scientific name	Family	Local name	Ha	Sources	Pu	Ethnic group	Medicinal uses against	Other local uses	Voucher number
*Allium cepa* L	Amaryllidaceae	Qulubi addi	H	Hg/Mt	Fb	G	Nasal bleeding, Passive sexual interest, Weight loss	Fo, Sp	St 2022 (1)
*Ananas comosus* (L.) Merr	Bromeliaceae	Anannase	H	Hg	Ff, Ffb	G	Skin infection	Fo, Env	St 2022 (2)
*Oldeania alpina* (K.Schum.) Stapleton	Poaceae	Lemma	Sh	Hg/Wl	As	G	Wound	Co, F, Fl, Env	St 2022 (3)
*Arundo donax* L	Poaceae	Hophetikka	Sh	Hg/Wl	Dl	G	Swellings	Co, F, Fl, Env	St 2022 (4)
*Bidens macroptera* (Sch.Bip. ex Chiov.) Mesfin	Asteraceae	Addeyi	H	Wl	Fr	G	Abnormal menstruation cycle, amoeba, diarrhea	Nt	St 2022 (5)
*Capsicum frutescens* L	Solanaceae	Mixmixxo	H	Hg/Mt	Ff	G	Amoeba, intestinal worms	Fo, Sp	St 2022 (6)
*Celtis africana* Burm.f	Cannabaceae	Shishu	T	Wl	Fb, Db, Or, Yfl, Yr, Fs	G	Stomachache, Jaundice, Skin infection, Wound, Headache, Diarrhea, Asthma, Intestinal worms, Glandular, Lung infection, Giardia	Fl, Co, Tm, Env, Sh, Ch, Fr	St 2022 (7)
*Clausena anisata* (Willd.) Hook.f. ex Benth	Rutaceae	Lichee/Limich	Sh	Wl	Fl	G	Swellings	Env, Fr	St 2022 (8)
*Clutia lanceolata* Forssk	Peraceae	Kudhure	Sh	Wl	Fl	G	Ear infection	Env, Fr, Fl	St 2022 (9)
*Colocasia esculenta* (L.) Schott	Araceae	Colcomma	H	Wl	Dr, Fr	G	Deep sores and Cancer-like ailments, Toothache	Env	St 2022 (10)
*Cymbopogon citratus* (DC.) Stapf	Poaceae	Hancura	H	Wl	Fl	G	Abortion, Bath of mother after giving birth, Blood pressure, Deep sores and Cancer-like ailments, Cholesterol, Kidney infection, Stomachache, Gonorrhea, Vomiting	Env	St 2022 (11)
*Dalbergia lactea* Vatke	Fabaceae	Batissa	Sh	Wl	Fl, Fs	G	Gonorrhea, Amoeba	Env, Fl, Fr	St 2022 (12)
*Drynaria volkensii* Heiron	Polypodiaceae	Bobile	Ep	Wl	Fr, Fl	G	Deep sores and cancer-like ailments, ear infection, swellings, nasal bleeding	Nt	St 2022 (13)
*Embelia schimperi* Vatke	Primulaceae	Honkoko	T	Wl	Fl	G	Glandular, Gonorrhea, Jaundice	Fl, Co	St 2022 (14)
*Euphorbia pulcherrima* Willd. ex Klotzsch	Euphorbiaceae	Qorsa abeba	Sh	Hg	Fr	G	Fever	F, Fl	St 2022 (15)
*Euphorbia tirucalli* L	Euphorbiaceae	Qinchibi	Sh	Hg	L	G	Deep sores and Cancer-like ailments	F, Fl	St 2022 (16)
*Fagaropsis angolensis* (Engl.) H.M.Gardner	Rutaceae	Sissa	T	Wl	Ds, Fl	G	Stomachache, Wound, Swellings	Fl, Env, Co	St 2022 (17)
*Flacourtia indica* (Burm.f.) Merr	Salicaceae	Hagala	Sh	Wl	Ff, Fr	G	Snake venom, Respiratory organ infection	F, Fl	St 2022 (18)
*Hibiscus macranthus* Hochst. ex A. Rich	Malvaceae	Abeba	Sh	Wl	Fl	G	Fire accident	F, Fl	St 2022 (19)
*Hyparrhenia rufa* (Nees) Stapf	Poaceae	Qoricha bekekko	H	Wl	Fl	G	Swellings, Cough, Lung infection	Env, Fr	St 2022 (20)
*Juniperus procera* Hochst. ex Endl	Cupressaceae	Honcho	T	Hg/Wl	Ds	G	Respiratory organ infection	Co, Tm, Fl, F	St 2022 (21)
*Kanahia laniflora* (Forssk.) R.Br	Asclepidaceae	Cigga	Sh	Wl	Fl	G	Jaundice	Fl	St 2022 (22)
*Leucas tomentosa* Gürke	Lamiaceae	Balbalato	H	Wl	Fl	G	Febrile illness	Env, Fl	St 2022 (23)
*Rubia cordifolia* L	Rubiaceae	Dummo	Cl	Wl	Fl	G	Malaria	Nt	St 2022 (24)
*Sesbania sesban* (L.) Merr	Fabaceae	Shashatto	Sh	Wl	Fl	G	Rabies, Snake venom	Env, Fr, Fl, Co	St 2022 (25)
*Sida ovata* Forssk	Malvaceae	Qirqixxe	Sh	Wl	Fl	G	Deep sores and Cancer-like ailments	Env, Fl	St 2022 (26)
*Solanum indicum* L	Solanaceae	Dimoxxa	Sh	Wl	Fl	G	Nasal bleeding, Skin infection, Snake venom	F, Fl	St 2022 (27)
*Sorghum bicolor* (L.) Moench	Poaceae	Xinqisha	H	Wl	Fr	G	Febrile illness, Respiratory organ infection	Fo	St 2022 (28)
*Strychnos spinosa* Lam	Loganiaceae	Goqqumma	T	Wl	Fl, Fb	G	Bad/evil spirit, Fire accident, Toothache	Fo, Env, Fl	St 2022 (29)
*Gymnanthemum myrianthum* (Hook.f.) H.Rob	Asteraceae	Rejii	Sh	Wl	Fl, Dr	G	Headache, Respiratory organ infection	Fr, Fl, Env, Co	St 2022 (30)
*Xanthium strumarium.* L	Asteraceae	Qorsi butika	H	Wl	Fl	G	Nerve case	Fl	St 2022 (31)
*Vachellia oerfota* (Forssk.) Kyal. & Boatwr	Fabaceae	Ajoo	Sh	Hg/Wl	Fr, Fb	O	Bad/evil spirit, General health	Co, Env, Fl, Hn, Fr, Ch, Sh, H	St 2022 (32)
*Vachellia seyal* (Delile) P.J.H.Hurter	Fabaceae	Waccu	T	Wl	Fb	O	Intestinal worms	Co, Env, Fl, Env, Co, Fr, Ch, Sh, Hn	St 2022 (33)
*Vachellia tortilis (*Forssk.) Galasso & Banfi	Fabaceae	Dhadacha	T	Wl	Fb	O	Malaria, Bad/evil spirit	Co, Env, Fl, Env, Co, Fr, Ch, Sh	St 2022 (34)
*Argemone mexicana* L	Papaveraceae	Wajota	H	Wl	L, Fl	O	Deep sores and Cancer-like ailments, Blood pressure, Wound, Jaundice	Fl	St 2022 (35)
*Beta vulgaris* L	Amaranthaceae	Keyisir	H	Hg/Mt	Fr, Fl	O	Anemia, Wound	Fo, Env	St 2022 (36)
*Calendula officinalis* L	Asteraceae	Olaati	H	Wl	Fs	O	Amoeba	Nt	St 2022 (37)
*Capsella bursa-pastoris* Medik	Brassicaceae	Bursi	H	Wl	Fr	O	Lung infection, Asthma, Cough	Env	St 2022 (38)
*Casimiroa edulis* La Llave	Rutaceae	Kazmiree	T	Hg	Ff	O	Gastric diseases	Fr, Fo, Fl, Co, Tm, Env, Ch	St 2022 (39)
*Casuarina equisetifolia* L	Casuarinaceae	Shawshawee	T	Wl	Fl	O	Rabies	Fl, Co	St 2022 (40)
*Citrus limon* (L.) Osbeck	Rutaceae	Lomme	T	Hg/Wl	Fl, Ff	O	Blood pressure, Fever, Stomachache, Common cold, Amoeba	Fo, Env	St 2022 (41)
*Rotheca myricoides* (Hochst.) Steane & Mabb	Lamiaceae	Marachissa	Sh	Wl	Fb, Fl	O	Rabies, Stomachache, Bad/evil spirit	Fr, Env	St 2022 (42)
*Cyathula polycephala* Baker	Amaranthaceae	Hixxicho	H	Wl	Fl	O	Febrile illness	Fl	St 2022 (43)
*Daucus carota* L	Apiaceae	Karotee	H	Hg/Mt	Fr, Ff	O	Jaundice, Passive sexual interest	Fo	St 2022 (44)
*Eleusine coracana* (L.) Gaertn	Poaceae	Dagussa	H	Wl	Fs	O	Bone injury, Wound	Fo	St 2022 (45)
*Eragrostis tef* (Zuccagni) Trotter	Poaceae	Gashee	H	Hg/Mt	Fs	O	Bone injury, Wound	Fo	St 2022 (46)
*Erica arborea* L	Ericaceae	Satto	Sh	WL	Fr, Dr, Dl	O	Malaria, Bad/evil spirit, Wound	Fl	St 2022 (47)
*Corymbia citriodora* (Hook.) K.D.Hill & L.A.S.Johnson	Myrtaceae	Bargamo sayiti	T	Hg/Wl	Fl	O	Gonorrhea	Co, Tm, F, Fl, Ch	St 2022 (48)
*Ficus sycomorus* L	Moraceae	Odda	T	Wl	Ds, Db, Fs	O	Tonsillitis, Glandular	Fl, Co, Env, Sh	St 2022 (49)
*Helianthus annuus* L	Asteraceae	Suffa	H	Hg/Mt	Fs	O	Febrile illness, Tung infection	Fo	St 2022 (50)
*Indigofera arrecta* Hochst. ex A.Rich	Fabaceae	Hinna	Sh	Wl	Fl	O	General health	Fr, Env, Fl	St 2022 (51)
*Kalanchoe densiflora* Rolfe	Crassulaceae	Hanculule Ancura	H	Wl	Fl	O	Muscular/joint pain	Nt	St 2022 (52)
*Kniphofia foliosa* Hochst	Asphodelaceae	Shushune	Sh	Wl	Fr	O	Stomachache	Nt	St 2022 (53)
*Lantana camara* L	Verbenaceae	Qoso jarti	Sh	Wl	Fl	O	Sneezing	Fl, F	St 2022 (54)
*Lippia abyssinica* (Otto & A.Dietr.) Cufod	Verbenaceae	Sukayi	H	Wl	Dl	O	Blood pressure, Diarrhea, Stomachache	Env, Fl, Sp	St 2022 (55)
*Gymnosporia senegalensis* (Lam.) Loes	Celastraceae	Kombolcha	Sh	Hg/Wl	Fb	O	Jaundice, Malaria, Skin infection	Fl, Ch, Co	St 2022 (56)
*Mimusops kummel* Bruce ex A.DC	Sapotaceae	Olaatee	T	Wl	Fs, Ds	O	Diarrhea, Lung infection	Fl, Co, Fr	St 2022 (57)
*Myrica salicifolia* Hochst. ex A.Rich	Myricaceae	Qammo	T	Wl	Fb	O	Bad/evil spirit	Fl, Co	St 2022 (58)
*Pavonia urens* Cav	Malvaceae	Hincinnii	H	Wl	Fl	O	Bad/evil spirit	Nt	St 2022 (59)
*Persicaria senegalensis* (Meisn.) Soják	Polygonaceae	Shulta	H	Wl	Fl	O	Jaundice, Malaria	Nt	St 2022 (60)
*Pittosporum viridiflorum* Sims	Pittosporaceae	Harbu	T	Wl	Fl, Fb	O	Rabies, Fever, Bad/evil spirit	Fl, Co	St 2022 (61)
*Plantago lanceolata* L	Plantaginaceae	Qorxxo	H	Wl	Fr	O	Epilepsy	Fr, Env	St 2022 (62)
*Aningeria altissima* (A.Chev.) Aubrév. & Pellegr	Sapotaceae	Kore	T	Wl	Fb	O	Swellings	Fl, Co, Env, Ch	St 2022 (63)
*Rubus apetalus* Poir	Rosaceae	Goorra	Sh	Wl	Yb, Fs	O	Toothache	Fo, F, Env	St 2022 (64)
*Rubus steudneri* Schweinf	Rosaceae	Goorra	Sh	Wl	Db, Fb, Fr, Fl	O	Headache, Nasal bleeding, Skin infection, Amoeba, Diarrhea, Urinary organ infection, Febrile illness, Stomachache	F, Fl	St 2022 (65)
*Salvia nilotica* Juss. ex Jacq	Lamiaceae	Hulegebi	H	Wl	As	O	Heart case	Env	St 2022 (66)
*Schinus molle* L	Anacardiaceae	Qondo	T	Wl	Fl, Fs, Yb	O	Jaundice, Tonsillitis, Nasal bleeding	Fl, Co, Fr, Sh	St 2022 (67)
*Schrebera alata* (Hochst.) Welw	Oleaceae	Dhamma’e	T	Wl	Fr	O	Deep sores and Cancer-like ailments, Swellings	Sh, Co, F, Fl, Env	St 2022 (68)
*Senna auriculata* (L.) Roxb	Fabaceae	Ajawa	Sh	Wl	Dr, Fl	O	Constipation, Skin infection	Fl, Fr, Env	St 2022 (69)
*Solanum marginatum* L.f	Solanaceae	Hidhi oromo	Sh	Wl	Fr, Ff, Fl	O	Febrile illness, Acid injury, Nasal bleeding, Snake venom, Autism, Bad/evil spirit	Fl, F	St 2022 (70)
*Vepris nobilis* (Delile) Mziray	Rutaceae	Hadhessa	T	Wl	Fl, Dl, Fr	O	Blood pressure, Skin infection, Dry skin treatment, Ear infection, Eye infection	Co, Env, Fl, Fr	St 2022 (71)
*Terminalia brownii* Fresen	Combretaceae	Rukessa	Sh	Hg	Fl	O	Common cold, Headache	Fl, Sh, Env	St 2022 (72)
*Trichilia dregeana* Sond	Meliaceae	Sissa	T	Wl	Ds, Fl	O	Jaundice	Fr, Co, Env, Sh	St 2022 (73)
*Ximenia americana* L	Olacaceae	Hudha	Sh	Wl	Fs, Fr	O	Swellings, Intestinal worms, Wound, Stomachache	Fo, Fl	St 2022 (74)
*Zea mays* L	Poaceae	Badala	H	Hg	Ds	O	Sneezing	Fo, Fl, Fr	St 2022 (75)
*Ziziphus spina-christi* (L.) Willd	Rhamnaceae	Qurqura	T	Wl	Fl, Fr, Ds, L, Fb	O	Skin infection, Bad/evil spirit, Rabies, Giardia, Gonorrhea, Eye infection, Intestinal worms, Wound	Co, Fo, Fr, Env, Sh, Ch	St 2022 (76)
*Aloe pirottae* A.Berger	Asphodelaceae	Sibri (G) Hargessa (O)	H	Wl	Fl, Dl	O, G	Gastric diseases, Jaundice, Kidney infection, Menstruation cycle disorder, Passive sexual interest, Vaginal infection, Ear infection	Fl, Env	St 2022 (77)
*Asparagus africanus* Lam	Asparagaceae	Uffae (G) Siriitii (O)	Sh	Wl	Fb, Fl, Ds	O, G	Rabies, Deep sores and Cancer-like ailments, Jaundice, Ear infection, Skin infection, Epilepsy, Swellings, Lung infection	Nt	St 2022 (78)
*Brassica carinata* A.Braun	Brassicaceae	Shaaana (G) Danqalle (O)	H	Hg	Fl, Ds	O, G	Constipation, Fever, Skin infection, Toothache, Cough, Lung infection	Fo, Env	St 2022 (79)
*Commelina benghalensis* L	Commelinaceae	Butikka (G) Lalunxe (O)	H	Wl	Fst, L	O, G	Swellings, Amoeba, Skin infection	Env	St 2022 (80)
*Delonix elata* (L.) Gamble	Fabaceae	Harangama (G) Sukeelaa (O)	Sh	Wl	Ds, Fs	O, G	Stomachache	F, Fl, Co, Env	St 2022 (81)
*Euphorbia ampliphylla* Pax	Euphorbiaceae	Caree (G) Surre (O)	Sh	Hg	Fr, L, Dr	O, G	Epilepsy Deep sores and Cancer-like ailments, Bad/evil spirit	F, Fr	St 2022 (82)
*Grewia ferruginea* Hochst. ex A.Rich	Malvaceae	Ogomodi (G) Dhoqona (O)	Sh	Wl	Fb, Fl, Dl, Fr, Fs	O, G	Respiratory organ infection, Jaundice, Febrile illness, Headache, Swellings, Wound, Epilepsy, Deep sores and Cancer-like ailments, Amoeba	Fl, Co, Env	St 2022 (83)
*Hagenia abyssinica* (Bruce) J.F.Gmel	Rosaceae	Kosso (G) Hexxo (O)	T	Wl	Ds, Fb, Fr	O, G	Tapeworms, Amoeba, Diarrhea, Gonorrhea, Febrile illness, Intestinal worms	Tm, Env, Ch	St 2022 (84)
*Hordeum vulgare* L	Poaceae	Dinae (G) Hayixxe (O)	H	Hg/Mt	Fs, Ds	O, G	Bone injury, Wound, Lightning	Fo, Env	St 2022 (85)
*Lepidium sativum* L	Brassicaceae	Fexxo (G) Sinfa (O)	H	Hg/Mt	Ds, Fr	O, G	Common cold, Febrile illness, Malaria, Vaginal infection, Dry skin treatment, Gastric diseases	Nt	St 2022 (86)
*Maesa lanceolata* Forssk	Primulaceae	Kagaye (G) Abbaye (O)	Sh	Wl	Fr, Fl, Ds, Fb	O, G	Jaundice, Nerve case, Muscular/joint pain, Skin infection, Gastric diseases, Ear infection, Amoeba, Gonorrhea, Cough	Fl, Fr, Co, Env	St 2022 (87)
*Nuxia congesta* R.Br. ex Fresen	Stilbaceae	Burcana (G,O)	T	Wl	Db, Fb	O, G	Deep sores and Cancer-like ailments, Skin infection, Wound	Co, Fl, Env, Ch	St 2022 (88)
*Ocimum gratissimum* L	Lamiaceae	Damakase (G) Qoricha michi (O)	Sh	Hg/Wl	Fl	O, G	Febrile illness, Fever, Eye infection, Vomiting, Malaria, Stomachache, Amoeba, Kidney infection	Fl	St 2022 (89)
*Prunus africana* (Hook.f.) Kalkman	Rosaceae	Garbicho (G,O)	T	Wl	Fb, Fs	O, G	Skin infection, Glandular, Goiter	Tm, Co, Fl, Fr, Ch, Sh	St 2022 (90)
*Psydrax schimperianus* (A.Rich.) Bridson	Rubiaceae	Dibexxo (G) Gallo (O)	T	Wl	Fb, Fl, Yfl	O, G	Blood pressure, Febrile illness, Skin infection, Deep sores and Cancer-like ailments, Wound, Muscle pain, Muscular/joint pain	Fl, Co, Env	St 2022 (91)
*Searsia pyroides* (Burch.) Moffett	Anacardiaceae	Dobossa (G) Dobobessa (O)	Sh	Wl	Fl, Fs, Ds, Dr	O, G	Deep sores and Cancer-like ailments, Autism, Passive sexual interest, Common cold, Bad/evil spirit, Epilepsy	Fl, Env	St 2022 (92)
*Sida schimperiana* Hochst. ex A.Rich	Malvaceae	Gebresso (G) Koti jebessa (O)	Sh	Wl	Fr, Fb, Dl, Db, Fl, Dr	O, G	Swellings, Gonorrhea, Headache, Lung infection, Toothache, Wound, Fever, Jaundice, Glandular	Env, Fl	St 2022 (93)
*Solanecio gigas* (Vatke) C.Jeffrey	Asteraceae	Dumbolla (G) Yeshikoko gomen (O)	Sh	Wl	Fs, Fl, Yr, Ds, Yfl, Fr	O, G	Lung infection, Gastric diseases, Jaundice, Malaria, Swellings, Amoeba, Diarrhea, Nasal bleeding, Glandular, Fever	Fl, Env	St 2022 (94)
*Aframomum corrorima* (A.Braun) P.C.M.Jansen	Zingiberaceae	Janjiwello	H	Hg/Mt	Ds	S	Skin infection, Tonsillitis	Sp	St 2022 (95)
*Antiaris toxicaria* (J.F.Gmel.) Lesch	Moraceae	Dimbicho	T	Wl	Fl, Fb	S	Rabies	Fl, Co	St 2022 (96)
*Artemisia absinthium* L	Asteraceae	Arity	H	Wl	Fl	S	Diabetes, Bad/evil spirit	Nt	St 2022 (97)
*Commelina africana* L	Commelinaceae	Lalunxe	H	Wl	Fl, L	S	Skin infection	Nt	St 2022 (98)
*Coriandrum sativum* L	Apiaceae	Dimbilale	H	Hg/Wl	Ds	S	Overall health	Sp	St 2022 (99)
*Cucumis dipsaceus* Ehrenb. ex Spach	Cucurbitaceae	Basu baqula	Cl	Wl	Ff	S	Jaundice	Nt	St 2022 (100)
*Cucumis prophetarum* L	Cucurbitaceae	Basu baqula	Cl	Hg/Wl	Ff, Fr, Ds, Dr	S	Deep sores and Cancer-like ailments, Amoeba, Diarrhea, Lung infection, Jaundice, Rheumatic, Balanced diet, Glandular, Respiratory organ infection	Nt	St 2022 (101)
*Cynodon dactylon* (L.) Pers	Poaceae	Qorcisha	H	Wl	Fst	S	Swellings	Env	St 2022 (102)
*Cynoglossum coeruleum* Hochst. ex A.DC	Boraginaceae	Hifaticho	H	Wl	Fl, Drh, Fr	S	Skin infection, Lung infection	Fl	St 2022 (103)
*Dovyalis caffra* (Hook.f. & Harv.) Warb	Salicaceae	Faranjete shisho	Sh	Hg/Wl	Fb	S	Snake venom	F, Fo, Fl, Co	St 2022 (104)
*Echinops kebericho* Mesfin	Asteraceae	Kebericho	H	Wl	Dr, Fr	S	Common cold, Febrile illness, Headache, Fever	Nt	St 2022 (105)
*Euclea racemosa subsp. schimperi* (A.DC.) F.White	Ebenaceae	Mi’essa	Sh	Wl	Db	S	Stomachache	Fr, Fl	St 2022 (106)
*Vicia lens* (L.) Coss. & Germ	Fabaceae	Misirra	H	Hg/Mt	Ds, Fs	S	Chickenpox, Spider poison, Wound	Fo	St 2022 (107)
*Lippia javanica* (Burm.f.) Spreng	Verbenaceae	Hanasho	Sh	Wl	Fl	S	Blood pressure	Fl, Sp, Fr	St 2022 (108)
*Momordica boivinii* Baill	Cucurbitaceae	Kiree	Cl	Wl	Fr, Fl, Ff, Ds	S	Bad/evil spirit, Lung infection, Jaundice, Stomachache, Toothache, Amoeba	Nt	St 2022 (109)
*Premna schimperi* Engl	Lamiaceae	Uddo	Sh	Wl	Fr, Fl	S	Lung infection, Febrile illness	Fl	St 2022 (110)
*Searsia natalensis* (Bernh. ex Krauss) F.A.Barkley	Anacardiaceae	Dawowesa	T	Wl	Fb, Or, Fl, Fs	S	Snake venom	Fl, Co, Sh, Env, Sh, Ch	St 2022 (111)
*Triticum turgidum subsp. dicoccum* (Schrank ex Schübl.) Thell	Poaceae	Ajja	H	Wl	Fs	S	Wound	Env, Fo	St 2022 (112)
*Ajuga integrifolia* Buch.-Ham. ex D.Don	Lamiaceae	Amessa (S) Anamuro (G)	H	Wl	Fl	S, G	Pain relief, Anemia, Stomachache, Malaria, Weight loss	Env	St 2022 (113)
*Capsicum annuum* L	Solanaceae	Mixmixxa (S) Mixmixxo (G)	H	Hg/Mt	Ff, Fl	S, G	Intestinal worms, Anemia, Common cold, Tonsillitis	Fo, Sp	St 2022 (114)
*Cinnamomum verum* J.Presl	Lauraceae	Kerefa (S) Kereffoe (G)	T	Wl	Db	S, G	Asthma, Common cold, Fever	Tm, Fl, Sh, Co, Env, Ch	St 2022 (115)
*Cucurbita pepo* L	Cucurbitaceae	Baqulla (S) Buqqee (G)	Cl	Hg/Mt	Ds	S, G	Tapeworms, Intestinal worms, Amoeba	Env	St 2022 (116)
*Ensete ventricosum* (Welw.) Cheesman	Musaceae	Wesse (S) Werqqoo (G)	H	Hg	Fl, Yfl	S, G	Lightning, Swellings, Amoeba, Gastric diseases	Env, Fo	St 2022 (117)
*Galinsoga quadriradiata* Ruiz & Pav	Asteraceae	Qorcisha (S) Qoricha (G)	H	Wl	Frw	S, G	Goiter, Tonsillitis, Toothache, Cancer, Swellings	Env	St 2022 (118)
*Impatiens ethiopica* Grey-Wilson	Balsaminaceae	Enshoshila (S) Abebba (G)	H	Wl	Fr, Fl	S, G	Gonorrhea	Nt	St 2022 (119)
*Linum usitatissimum* L	Linaceae	Telba (S) Telibao (G)	H	Hg/Wl	Ds, Drh	S, G	Gastric diseases, Blood pressure, Diabetes, Weight loss, Kidney infection, Cough, Lung infection, Tuberculosis	Fr, Env, Fo	St 2022 (120)
*Coleus igniarius* Schweinf	Lamiaceae	Tontona (S) Tontonammo (G)	Sh	Wl	Fl, Fr, Drh	S, G	Amoeba, Skin infection, Febrile illness, Bad/evil spirit, Wound, Stomachache, Intestinal worms	Fl, Fr, Env	St 2022 (121)
*Solanum nigrum* L	Solanaceae	Xunayee (S) Awuxxi (G)	Sh	Wl	Fl, S, F	S, G	Stomachache	Fo, Fr	St 2022 (122)
*Rumex nepalensis* Spreng	Polygonaceae	Tulte (S) Gangago (G)	H	Wl	Fr, Dl	S, G	Stomachache, Intestinal worms, Wound	Env	St 2022 (123)
*Saccharum officinarum* L	Poaceae	Shaonkora (S) Sukari (G)	H	Hg	Fst	S, G	Gastric diseases	Fo	St 2022 (124)
*Thymus schimperi* Ronniger	Lamiaceae	Tosign (S) Sogetti (G)	H	Wl	Dl, Fl	S, G	Blood pressure, Cholesterol, Bad/evil spirit	Sp, Env	St 2022 (125)
*Trigonella foenum-graecum* L	Fabaceae	Shiqoo (S) Shiqoe (G)	H	Hg/Wl	Ds, Fs	S, G	Cholesterol, Blood pressure, Kidney infection, Cough, Lung infection, Tuberculosis, Abnormal menstruation cycle, Weight loss, Gastric diseases, Loss of appetite, Menstruation cycle disorder, Stomachache	Sp	St 2022 (126)
*Vicia faba* L	Fabaceae	Attarra (S) Baqello (G)	H	Hg	Fs	S, G	Gastric diseases	Fo, Fr	St 2022 (127)
*Aloe vera* (L.) Burm.f	Asphodelaceae	Argissa (S) Algae (O)	H	Hg/Wl	Fl, L	S, O	Amoeba, Malaria, Blood pressure, Stomachache	Fl	St 2022 (128)
*Balanites aegyptiaca* (L.) Delile	Zygophyllaceae	Gidicho (S) Bedenno (O)	T	Wl	Ds, Fb, Fs, Dst	S, O	Amoeba, Diarrhea, Stomachache, Mental case, Headache	Fl, Co, Fr, Sh	St 2022 (129)
*Carissa spinarum* L	Apocynaceae	Gora (S) Hagamssa (O)	Cl	Wl	Fr, Fs, Fb, Fl	S, O	Gonorrhea, Diarrhea, Bad/evil spirit, Febrile illness, Headache	Nt	St 2022 (130)
*Dodonaea viscosa subsp. angustifolia* (L.f.) J.G.West	Sapindaceae	Itancha (S) Xidacha (O)	Sh	Wl	Fl, Fb, Fr	S, O	Lung infection, Headache, Bone injury, Circumcision wound, Gastric diseases, Stomachache	Fl, Fr, Env, Co	St 2022 (131)
*Euclea divinorum* Hiern	Ebenaceae	Mi’essa (S) Miessa (O)	Sh	Wl	Fr, Dl, Fl, Fb	S, O	Intestinal worms, Skin infection, Weight loss, Circumcision wound	Fl	St 2022 (132)
*Mentha spicata* L	Lamiaceae	Nana (S) Naana (O)	H	Wl	Fl, Dl	S, O	Blood pressure	Sp	St 2022 (133)
*Ocimum jamesii* Sebald	Lamiaceae	Ambibisha (S) Hancabii (O)	Sh	Wl	Fl	S, O	Febrile illness, Worms	Fl	St 2022 (134)
*Persea americana* Mill	Lauraceae	Abukato (S) Abokaato (O)	T	Hg	Ds	S, O	Passive sexual interest, Blood pressure, Amoeba	Fo, Fl, Co, Sh, Fr, Ch	St 2022 (135)
*Pittosporum abyssinicum* Delile	Pittosporaceae	Boncho (S) Bobanticha (O)	T	Wl	Fl, Fb	S, O	Tuberculosis, Intestinal worms	Co, Fl, Fr	St 2022 (136)
*Rhamnus prinoides* L'Hér	Rhamnaceae	Xaaddo (S) Geshoo (O)	Sh	Hg	Yb, Or, Fl, Fr, Fs	S, O	Tonsillitis, Gonorrhea, Skin infection, Stomachache	Fl, Fr	St 2022 (137)
*Searsia glutinosa* (Hochst. ex A.Rich.) Moffett	Anacardiaceae	Oloncho (S) Olonchissaa (O)	T	Wl	Fl, Fr, Db, Fb	S, O	Lung infection, Bad/evil spirit, Glandular	Co, Tm, Sh, Fl, Fr, Sh, Ch	St 2022 (138)
*Rumex abyssinicus* Jacq	Polygonaceae	Shishone (S) Dhangogo (O)	H	Wl	Fr, Fb	S, O	Skin infection, Gastric diseases, Amoeba, Deep sores and Cancer-like ailments	Nt	St 2022 (139)
*Solanum incanum* L	Solanaceae	Borbodho (S) Hidhi loni (O)	Sh	Wl	Fl, Fr	S, O	Nasal bleeding, Snake venom, Bad/evil spirit, Diarrhea	Fl, F	St 2022 (140)
*Taverniera abyssinica* A.Rich	Fabaceae	Dingatagna (S) Dingataagnaa (O)	Sh	Wl	Drh, Fr, Dst	S, O	Febrile illness, Fever, Headache	Nt	St 2022 (141)
*Zehneria scabra* (L.f.) Sond	Cucurbitaceae	Abajole (S) Aba ejole (O0	Cl	Wl	Ds, Fl, Fr	S, O	Cancer, Gastric diseases, Bad/evil spirit, Swellings	Nt	St 2022 (142)
*Achyranthes aspera* L	Amaranthaceae	Cikicho (S) Maxxane (O) Derguu (G)	H	Wl	Fr, Fl	S, O, G	Gonorrhea, Stomachache, Headache, Muscle pain, Joint pain, Deep sores and Cancer-like ailments, Skin infection, Diarrhea, Respiratory organ infection, Bad/evil spirit, Jaundice, Lung infection, Ear infection, Nerve case	Env	St 2022 (143)
*Acokanthera schimperi* (A.DC.) Benth. & Hook.f. ex Schweinf	Apocynaceae	Qararo (S) Qararu (O) Dumugaa (G)	Sh	Wl	Ds, Fl, Fs	S, O, G	Bad/evil spirit, Skin infection, Wound	Fl, Sh, Fo, Env, Co	St 2022 (144)
*Albizia gummifera* (J.F.Gmel.) C.A.Sm	Fabaceae	Maticho (S) Mukarbaa (O) Gorbe (G)	T	Hg/Wl	Fb, Db, Fl, Fr	S, O, G	Deep sores and Cancer-like ailments, Goiter, Toothache, Dizziness, Stomachache, Jaundice, Lung infection, Amoeba, Malaria, Fire accident, Skin infection, Epilepsy, Febrile illness, Glandular, Gonorrhea, Swellings, Fever, Bad/evil spirit, Cough, Tuberculosis, Menstruation cycle disorder, Typhoid, Intestinal worms	Tm, Sh, Env, Fl, Fr, Co, Ch	St 2022 (145)
*Allium sativum* L	Amaryllidaceae	Wajjo tuma (S) Qulubii adi (O) Dimoxxa Sunkurta (G)	H	Hg/Mt	Fbb	S, O, G	Common cold, Malaria, Typhoid, Fever, Headache, Febrile illness, Gonorrhea, Chicken pox, Tonsillitis, Blood pressure, Skin infection, Stomachache, Asthma, Tung infection	Sp	St 2022 (146)
*Aloe macrocarpa* Tod	Asphodelaceae	Argissa (S) Hargissa (O) Algae (G)	H	Wl	L, Fl, Dl	S, O, G	Malaria, Jaundice, Typhoid, Fever, Deep sores and Cancer-like ailments, Lung infection, Gonorrhea, Pain relief, Urinary organ infection, Intestinal worms, Wound, Stomachache, Diarrhea	Fl	St 2022 (147)
*Artemisia abyssinica* Sch.Bip. ex Oliv. & Hiern	Asteraceae	Ciqugn (S) Qoricha (O) Sugeete (G)	H	Wl	Fl	S, O, G	Bad/evil spirit, Blood pressure, Malaria, Nasal bleeding, Chicken pox, Febrile illness, Skin infection, Bath of mother after giving birth, Headache	Env	St 2022 (148)
*Bersama abyssinica* Fresen	Francoaceae	Xewerako (S) Lolichissa (O) Jejeba (G)	T	Wl	Yb, Fl, Fs, Db, Fb, Yfl	S, O, G	Jaundice, Bad/evil spirit, Amoeba, deep sores and Cancer-like ailments, Intestinal worms, skin infection, Stomachache, Lung infection	Co, Fl, Fr, Env	St 2022 (149)
*Brucea antidysenterica* J.F.Mill	Simaroubaceae	Laffa (S) Abalcho (O) Waginos (G)	T	Wl	Fs, Ds, Db, Fb	S, O, G	Gonorrhea, Diarrhea, Stomachache	Tm, Co, Fl, Sh, Env, Fr	St 2022 (150)
*Calpurnia aurea* (Aiton) Benth	Fabaceae	Cekatta (S) Cekatta/ceqaa (O) Luxxa (G)	Sh	Wl	Fl, Fs, Ds, Dr, Fr, Fb, Dl, Dst	S, O, G	Lung infection, Typhoid, Intestinal worms, Jaundice, Bad/evil spirit, Amoeba, Headache, Stomachache, Toothache, Fever, Skin infection, Wound, Circumcision wound, Febrile illness, Deep sores and Cancer-like ailments, Deep sores and Cancer-like ailments, Swellings, Glandular, Respiratory organ infection	Fr, Env, Co, Fl,	St 2022 (151)
*Carica papaya* L	Caricaceae	Papaye (S) Papayee (O) Papayee (G)	T	Hg	Ds, Fs, Ff, Fl, L	S, O, G	Malaria, Intestinal worms, Gastric diseases, Fever, Bath of mother after giving birth, Blood pressure, Deep sores and Cancer-like ailments, Typhoid, Skin infection	Fo	St 2022 (152)
*Catha edulis* (Vahl) Forssk. ex Endl	Celastraceae	Catte (S, O, G)	Sh	Hg	Fr, Fl, Yfl, Or	S, O, G	Amoeba, Depression, Gonorrhea, Bad/evil spirit, Skin infection, Diarrhea	Fl, Co, Fr	St 2022 (153)
*Citrus* × *aurantiifolia* (Christm.) Swingle	Rutaceae	Qomxaxxe (S) Burtukanne (O, G)	Sh	Hg	Ds, Ff	S, O, G	Amoeba, Anemia, Blood pressure, Giardia	Fo, Fl	St 2022 (154)
*Clematis hirsuta* Perr. & Guill	Ranunculaceae	Fidhe (S) Fidhe Fittii (O) Labbicha (G)	Cl	Wl	Fr, Fl	S, O, G	Jaundice, Deep sores and Cancer-like ailments, Wound, Ear infection	Nt	St 2022 (155)
*Clutia abyssinica* Jaub. & Spach	Peraceae	Binjile (S) Ullefoni (O) Binjiloo (G)	H	Wl	Fr, Fs, Wp, Fl, L	S, O, G	Deep sores and Cancer-like ailments, Diarrhea, swellings, Spiritual, Snake venom, Wound, Toothache	Nt	St 2022 (156)
*Coffea arabica* L	Rubiaceae	Bunna (S) Buna (O) Buno (G)	Sh	Hg/Mt	Ds, Dl, Fl, Fb, Ffb	S, O, G	Gastric diseases, Malaria, Wound, Sneezing, Kidney infection, Swellings, Jaundice, Deep sores and Cancer-like ailments, Toothache, Depression	Fo, Fr, Co, Fl	St 2022 (157)
*Cordia africana* Lam	Boraginaceae	Wadicho (S) Wodessa (O) Wadissa (G)	T	Hg/Wl	Fb, Fs, Ds	S, O, G	Nerve case, Passive sexual interest, Bad/evil spirit, Blood pressure, Diarrhea, Deep sores, and Cancer-like ailments	Co, Fl, Fr, Sh, Tm, Env, Fo, Ch. Hn	St 2022 (158)
*Croton macrostachyus* Hochst. ex Delile	Euphorbiaceae	Masinna (S) Makkonissa (O) Mokonissa (G)	T	Hg/Wl	Fb, L, Fl, Yb, Db, Fr, Dl, Ds, Fs, L, Or	S, O, G	Deep sores and Cancer-like ailments, Eye infection, Lightning, Tetanus, Lung infection, Gonorrhea, Dizziness, Febrile illness, Wound, Bad/evil spirit, Diarrhea, Jaundice, Amoeba, Glandular, Giardia, Abortion, Intestinal worms, Malaria, Asthma, Typhoid, Skin infection, Placental delay during birth, Circumcision wound, Stomachache, Ear infection, Allergy, Menstruation cycle disorder	Co, Fl, Fr, Sh, Tm, Env, Hn	St 2022 (159)
*Datura stramonium* L. test	Solanaceae	Banje (S) Asanjiraa (O) Atefarisse (G)	H	Wl	Fl, Ds, Fr	S, O, G	Skin infection, Head skin infection, Toothache, Rabies	Fl	St 2022 (160)
*Ehretia cymosa* Thonn	Boraginaceae	Gidincho (S) Ulaga (O) Suggate (G)	T	Wl	Fl, Fb, Fs	S, O, G	Wound, Nasal bleeding, Skin infection, Lung infection, Deep sores and Cancer-like ailments, Stomachache, Swellings	Co, Fl, Fr, Sh, Tm, Env	St 2022 (161)
*Ekebergia capensis* Sparrm	Meliaceae	Oloncho (S) Onnonna (O) Olonchissaa (G)	T	Wl	Ds, Fb, Fs, Fl, Drh, Rb, Db	S, O, G	Amoeba, Goiter, Jaundice, Gonorrhea, Tuberculosis, Typhoid, Fever, Stomachache, Deep sores and Cancer-like ailments, Placental delay during birth, Skin infection, Bad/evil spirit, Swellings, Wound, Glandular, Diarrhea, Febrile illness	Co, Fl, Fr, Sh, Tm, Env, Ch, Hn	St 2022 (162)
*Erythrina abyssinica* Lam	Fabaceae	Welako (S) Wallenu (O) Walenu (G)	T	Wl	Fb, Fs, Fl, Db	S, O, G	Toothache, Bad/evil spirit, Diarrhea, Rabies, Intestinal worms, Lung infection, Goiter, Fever, Malaria, Eye infection, Cough, Skin infection, Tuberculosis, Liver infection	Co, Fl	St 2022 (163)
*Eucalyptus globulus* Labill	Myrtaceae	Wajo barzafe (S) Bargamo addi (O) Dimmu barzafe (G)	T	Wl	Fl	S, O, G	Asthma, Common cold, Pain relief, Bath of mother after giving birth, Fever, Mental case, Headache, Dry skin treatment, Skin infection, Bad/evil spirit, Nerve case, Nasal bleeding, Amoeba	Co, Fl, Sh, Tm	St 2022 (164)
*Justicia schimperiana* (Hochst. ex Nees) T.Anderson	Acanthaceae	Cikicho (S) Gulbana (O) Dummugaa (G)	Sh	Wl	Fr, Fl, Or	S, O, G	Amoeba, Rabies, Gonorrhea, Stomachache, Sneezing, Jaundice, Ear infection, Glandular, Goiter, Malaria, Epilepsy	Fl, Env	St 2022 (165)
*Kalanchoe petitiana* A.Rich	Crassulaceae	Hanculule (S) Qorso hoxisso (O) Wundifo (G)	H	Wl	Fl, Fr	S, O, G	A broken bone, Pain relief, Muscular/joint pain, Glandular, Diarrhea, Bone injury	Env	St 2022 (166)
*Lactuca inermis* Forssk	Asteraceae	Ameessa (S) Anamurro (O) Anamurro (G)	H	Wl	Fl, Wp	S, O, G	Balanced diet, Weight loss, Anemia, Febrile illness, Stomachache	Nt	St 2022 (167)
*Lagenaria siceraria* (Molina) Standl	Cucurbitaceae	Surupha (S) Buqee (O) Boto (G)	Cl	Wl	Or, Fr, Ds, Ff, Fl	S, O, G	Lung infection, Jaundice, Glandular, Fever, Joint pain, Amoeba, Goiter, Pain relief	Nt	St 2022 (168)
*Melia azedarach* L	Meliaceae	Niimi (S) Kininin (O) Kinini (G)	T	Hg	Fb, Fl, Fs, Dr, Db	S, O, G	Diabetes, Malaria, Stomachache, Depression, Diarrhea, Blood pressure, Gastric diseases, Nasal bleeding, Pain relief, Jaundice, Toothache, Deep sores and Cancer-like ailments, Intestinal worms, Typhoid, Fever, Glandular, Breast cancer	Fl, Sh, Fo, Fr, Co	St 2022 (169)
*Millettia ferruginea* (Hochst.) Hochst. ex Baker	Fabaceae	Hengedicho (S) Birbiraa (O) Birbirro (G)	T	Wl	Fb, Fr, Fbb, Fl, Db	S, O, G	Amoeba, Gonorrhea, Typhoid, Skin infection, Blood pressure, Stomachache, Malaria, Deep sores and Cancer-like ailments, Jaundice, Toothache, Ear infection, Goiter, Lung infection, Pain relief	Co, Fl, Fr, Sh, Tm, Env, Hn	St 2022 (170)
*Moringa stenopetala* (Baker f.) Cufod	Moringaceae	Shiferaw (S, O, G)	T	Hg/Mt	Dl, Fb, Fl, Fr, Db	S, O, G	Blood pressure, Glandular, Jaundice, Malaria, Diarrhea, Kidney infection, Lung infection, Deep sores and Cancer-like ailments, Gastric diseases, Cholesterol, Nerve case, Pain relief, Intestinal worms, Typhoid	Fo, Fl, Co, Env, Fr	St 2022 (171)
*Nicotiana tabacum* L	Solanaceae	Araddo (S) Tambo (O) Tambo (G)	H	Hg	Dl	S, O, G	Headache, Wound, Depression, Common cold	Fl	St 2022 (172)
*Nigella sativa* L	Ranunculaceae	Wajjo azmude (S) Qoricha adi (O) Azmuddo (G)	H	Hg/Mt	Ds	S, O, G	Common cold, Respiratory organ infection, Febrile illness, Skin infection, Amoeba, Nasal bleeding, Fever, Malaria, Asthma, Pain relief, Stomachache, Nerve case, Bone injury, Deep sores and Cancer-like ailments	Sp	St 2022 (173)
*Ocimum lamiifolium* Hochst. ex Benth	Lamiaceae	Michete xagicho (S) Qorsa mich I (O) Damakase (G)	Sh	Wl	Fl, Fr	S, O, G	Headache, Malaria, Febrile illness, Fever, Stomachache, Muscular/joint pain, Amoeba, Gonorrhea, Typhoid, Diarrhea	Fl	St 2022 (174)
*Olea europaea subsp. cuspidata* (Wall. & G.Don) Cif	Oleaceae	Ejerissa (S) Ejerissa (O) Woyira (G)	T	Wl	Dst, Fs, Fl, Fb, Fr, Yp, Db	S, O, G	Deep sores and Cancer-like ailments, Skin infection, Wound, Anemia, Blood pressure, Cough, Malaria, Respiratory organ infection, Bad/evil spirit, Tuberculosis, Jaundice, Cancer, Swellings, Toothache, Kidney infection, Vaginal infection, Intestinal worms, Pain relief, Asthma	Co, Fl, Fr, Sh, Tm, Env, Ch	St 2022 (175)
*Olinia rochetiana* A.Juss	Penaeaceae	Noole (S) Gunna (O) Dimexxo (G)	T	Wl	Fl, Fb, Dl, Db	S, O, G	Stomachache, Glandular, Skin infection, Wound, Circumcision wound, Toothache, Deep sores and Cancer-like ailments, Tuberculosis	Co, Fl, Fr, Sh, Tm, Env, Ch	St 2022 (176)
*Phytolacca dodecandra* L'Hér	Phytolaccaceae	Haranjicho (S) Andodde (O) Indoode (G)	Sh	Wl	Fr, Fl, Or, Yr	S, O, G	Abortion, Amoeba, Intestinal worms, Gonorrhea, Swellings, Giardia, Stomachache, Skin infection	Fl	St 2022 (177)
*Afrocarpus falcatus* (Thunb.) C.N.Page	Podocarpaceae	Dagucho (S) Zigbaa (O) Zigbo (G)	T	Wl	Fl, Fb, Db	S, O, G	Gonorrhea, Typhoid, Malaria, Jaundice, Deep sores and Cancer-like ailments, Wound, Glandular, Toothache, Amoeba	Co, Fl, Fr, Sh, Tm, Env, Ch	St 2022 (178)
*Psidium guajava* L	Myrtaceae	Zaytone (S) Zaytunna (O) Zayitunne (G)	T	Hg	Fl, Dl	S, O, G	Blood pressure, Malaria, Diabetes, Stomachache, Deep sores and Cancer-like ailments, Intestinal worms, Typhoid	Co, Fl, Fo, Fr, Env, Ch	St 2022 (179)
*Ricinus communis* L	Euphorbiaceae	Qomboho (S) Qobboo (O) Gullo (G)	Sh	Hg	Fr, Ds, Yr, Fs	S, O, G	Jaundice, Lung infection, Swellings, Tonsillitis, Wound, Skin infection	Co, Fl, F	St 2022 (180)
*Ruta chalepensis* L	Rutaceae	Sunkurta (S) Ciradamma (O) Xenadame (G)	H	Hg	Fl, Fr, Frw, Dl	S, O, G	Gonorrhea, Typhoid, Febrile illness, Goiter, Tuberculosis, Skin infection, Diarrhea, Bad/evil spirit, Malaria, Vomiting, Nerve case, Dry skin treatment, Stomachache, Giardia, Jaundice, Nasal bleeding, Placental delay during birth, Epilepsy, Swellings, Asthma, Headache, Anemia, Glandular, Deep sores and Cancer-like ailments, Bath of mother after giving a birth, Menstruation cycle disorder	Nt	St 2022 (181)
*Stephania abyssinica* (Quart.-Dill. & A.Rich.) Walp	Menispermaceae	Kelala (S, O) Kelaalaa (G)	Cl	Wl	Fl, Fr	S, O, G	Jaundice, Glandular, Lung infection, Gonorrhea, Deep sores and Cancer-like ailments	Nt	St 2022 (182)
*Syzygium guineense* (Willd.) DC	Myrtaceae	Duwancho (S) Badessa (O) Baddessa (G)	T	Wl	Fb, Fl, Dr, Dl, Fr, Yr	S, O, G	Amoeba, Diarrhea, Muscle pain, Bad/evil spirit, Skin infection, Lung infection, Weight loss, Glandular, Circumcision wound, Pain relief, Swellings, Deep sores and Cancer-like ailments	Co, Fl, Fr, Sh, Tm, Env, Ch	St 2022 (183)
*Urtica dioica* L	Urticaceae	Sonicho (S) Lalesa (O) Sonno (G)	H	Wl	Fr, Dr	S, O, G	Amoeba, Bad/evil spirit, Gonorrhea, Febrile illness, Deep sores and Cancer-like ailments	Fl	St 2022 (184)
*Urtica simensis* Hochst. ex A.Rich	Urticaceae	Sonicho (S) Lalesa (O) Sonno (G)	H	Wl	Fl, Fr, Dr	S, O, G	Bad/evil spirit, Fire accident, Febrile illness, Gastric diseases, Amoeba, Intestinal worms, Stomachache	Fl	St 2022 (185)
*Gymnanthemum amygdalinum* (Delile) Sch.Bip	Asteraceae	Hecho (S) Ebicha (O) Ebicha (G)	Sh	Wl	Fl, Fr, Yr	S, O, G	Amoeba, Malaria, Skin infection, Stomachache, Diarrhea, Head skin infection, Gonorrhea, Rabies, Febrile illness, Intestinal worms, Gastric diseases, Lung infection, Blood pressure, Jaundice, Vomiting, Typhoid	Fr, Fl, Co, Env, Hn	St 2022 (186)
*Gymnanthemum auriculiferum* (Hiern) Isawumi	Asteraceae	Rejee (S) Rejii (O) Ebicha (G)	Sh	Wl	Fl, Fr	S, O, G	Bad/evil spirit, Snake venom, Bath of mother after giving birth	Fl, Fr, Co, Env	St 2022 (187)
*Withania somnifera* (L.) Dunal	Solanaceae	Bula (S) Kummo (O) Bulla (G)	Sh	Wl	Fl, Fb, Ds, Fr, Db, OrDst,	S, O, G	Bad/evil spirit, Asthma, Cough, Skin infection, Febrile illness	Fl, Co	St 2022 (188)
*Zingiber officinale* Roscoe	Zingiberaceae	Janjiwello (S) Zinjibilla (O) Jaanjibeloo (G)	H	Hg/Mt	Drh, Frh	S, O, G	Asthma, Blood pressure, Passive sexual interest, Common cold, Tonsillitis, Typhoid, Headache, Malaria, Wound, Fever, Cough, Tung infection, Goiter, Constipation, Febrile illness, Amoeba, Gastric diseases, Stomachache	Sp	St 2022 (189)

(Ha = Habit, T = Tree, Sh = Shrub, H = Herb, Cl = Climber, Ep = Epiphyte, Pu = Parts used, Fl = Fresh leaf, Yfl = Young fresh leaf, Dl = Dry leaf, Fs = Fresh seed, Ds = Dry seed, Fr = Fresh root, Dr = Dry root, Or = Old root, Yr = Young root, Fb = Fresh bark, Db = Dry bark, Ff = Fresh fruit, L = Latex, Fbb = Fresh bulb, Drh = Dry rhizome, Frh = Fresh rhizome, Yl = Young leaf, Yr = Young root, Ffb = Fresh fruit bark, As = Ash, Yb = Young bud, Fst = Fresh stolon, Frw = Fresh flower, Fst = Fresh stem, Drs = Dry stem, Wp = Whole parts, Rb Root bark, Yp = Young petiole, Fl = Fuel wood, Env = Environmental role, F = Fence, Sh = Shading, Ch = Charcoal, Tm = Timber, Fo = Food, Co = Construction, Fr = Fodder, Nt = Not mentioned, Sp = Spice, Hn = Honey production)

### Influences of socio-demographic variables on medicinal plant knowledge

**Fig. 2:**
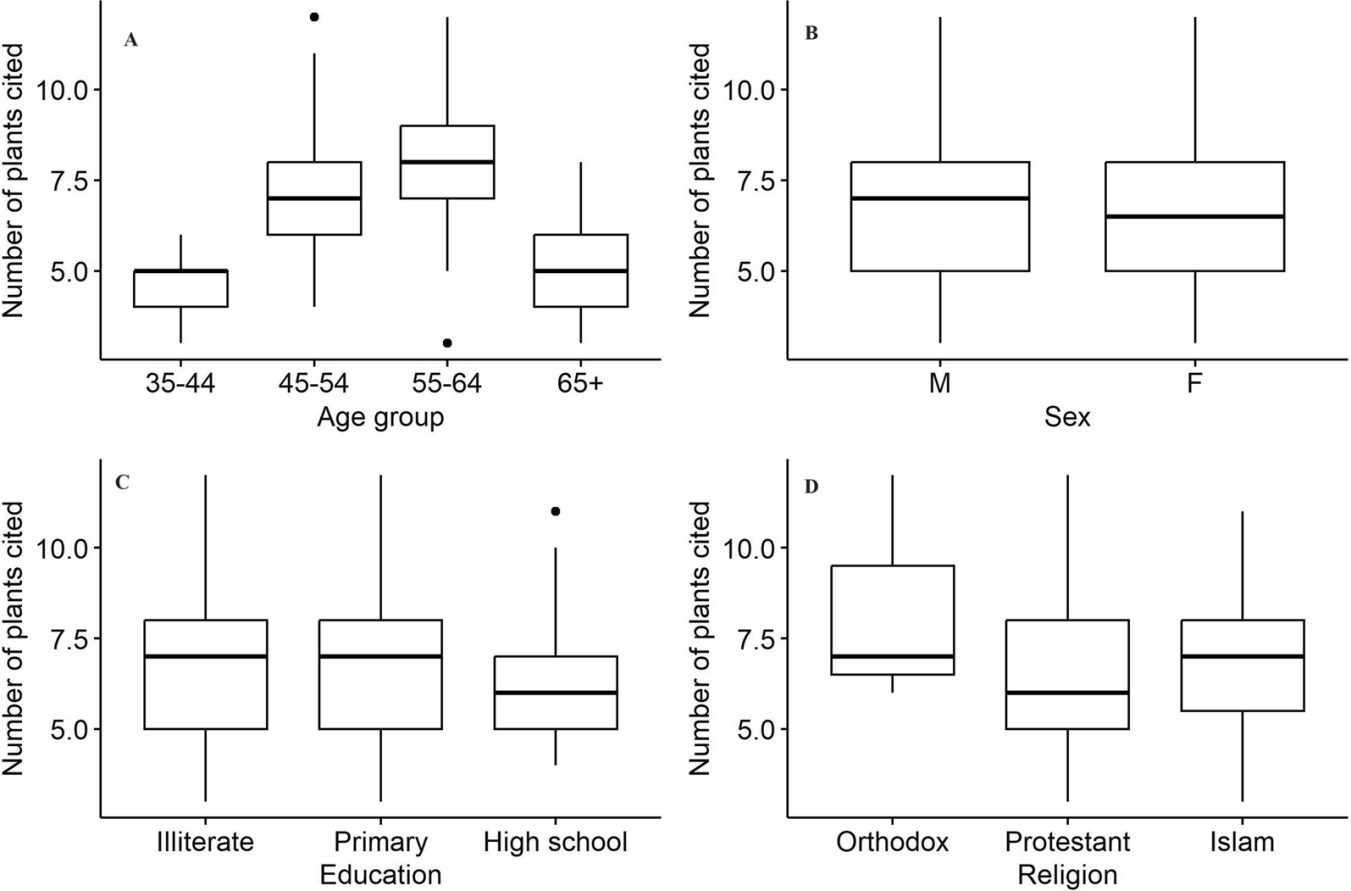
Average number of medicinal plants reported by each socio-demographic variables

The average number of medicinal plants reported by each socio-demographic variable was compared. It revealed that older traditional healers reported more medicinal plants (8) than others (Table 4 and Fig. 2A). Medicinal plants reported by followers of the Orthodox religion were higher (9) than Protestants and Muslims (Table 4 and Fig. 2D). The gender groups and education level categories reported a similar number of medicinal plants (7 each) (Table 4 and Fig. 2B).

**Table 4:** Comparison of the number of medicinal plants cited by different socio-demographic parameters across ethnic groups (Gedeo, Oromo, and Sidama)

Parameters	Categories	Number of informants (N = 189)	Mean ± SD	p-value
Age	Young (35–44)	33	4.73 ± 0.94	0.0001*
	Middle age (45–54)	45	6.84 ± 1.74	
	Older (55–64)	88	7.9 ± 1.72	
	Elderly (65 +)	23	5.09 ± 1.47	
Gender	Male	133	6.76 ± 2.02	0.77
	Female	56	6.73 ± 2.09	
Education	Illiterate	89	6.74 ± 2.03	0.54
	Primary level	81	6.86 ± 2.09	
	Secondary level	19	6.37 ± 1.89	
Religion	Protestant	101	6.51 ± 2.04	0.11
	Islam	75	7.04 ± 1.95	
	Orthodox	13	8.33 ± 3.21	

^*^Significance association (p < 0.05) between the averages of paired parameters

Our findings revealed a positive association between traditional healers' ethnobotanical knowledge and their ages (Kruskal–Wallis chi-squared = 84.375, df = 3, p-value = 0.0001) (Table 4). However, no significant association was found between the gender, education, and religion groups and ethnobotanical knowledge (p-value = 0.77, 0.54, and 0.11), respectively (Table 4).

### The correlation between ethnobotanical knowledge and age

**Fig. 3:**
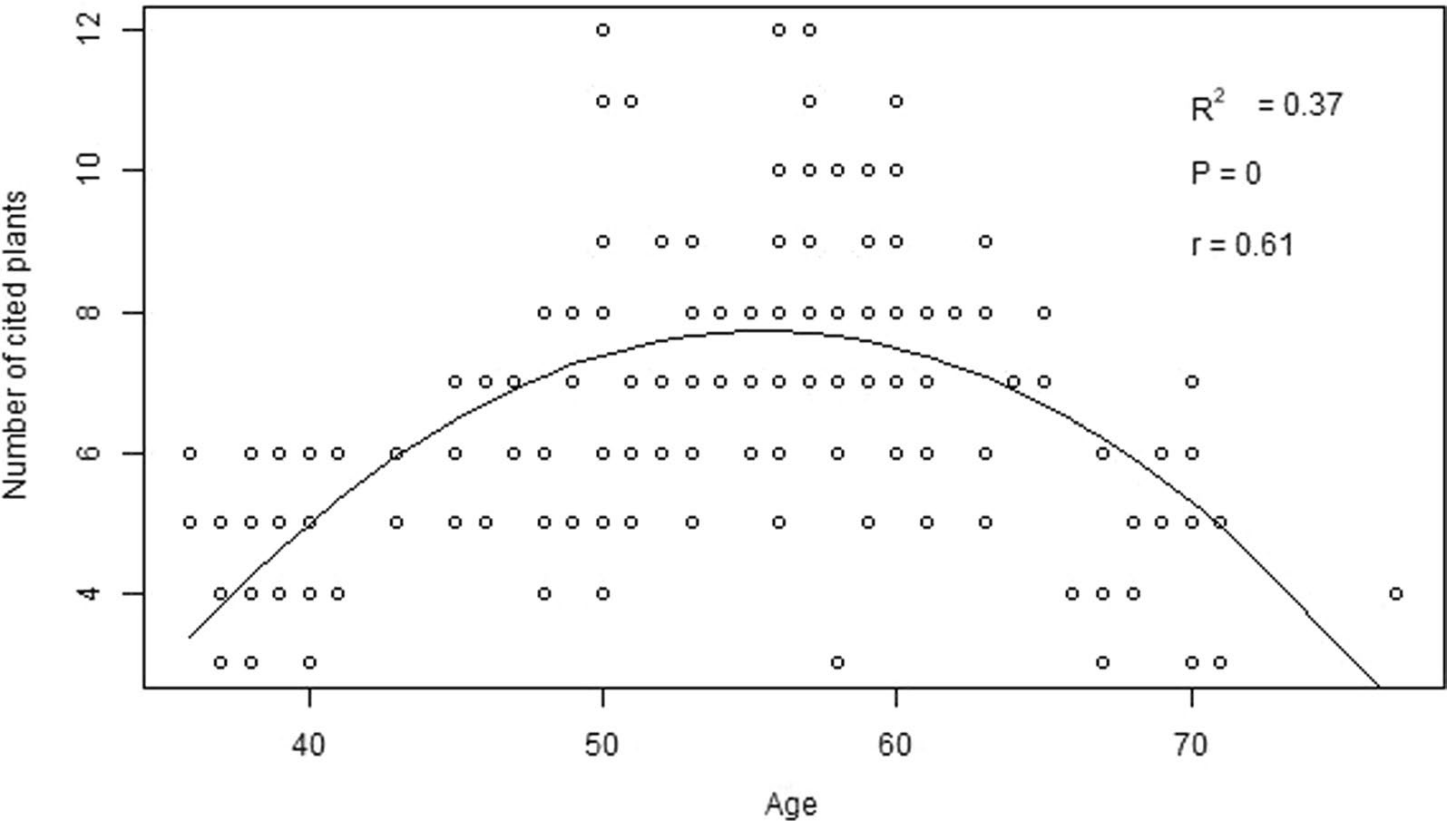
Correlation between the respondent's knowledge of medicinal plant citation and his or her age

The regression analyses across ethnic groups showed that the respondent's age is positively correlated with his/her knowledge of identifying medicinal plants used; however, it showed a curvilinear relationship at the end (Fig. 3). Overall, the influence of age accounted for 37% of the variation in knowledge of medicinal plants across ethnic groups.

### The most useful medicinal plant species of the studied ethnic groups

The use value index (UVI) is applied to measure various uses assigned to a specific plant species. Of the total 189 documented medicinal plants (Table 3), 78 medicinal plant species, which were claimed by three or more informants as remedies, were evaluated and revealed significant species use variation among the ethnic groups studied (Table 5). Meanwhile, 24 medicinal plant species scored the lowest use value; others were moderate to highest value (Table 5).

**Table 5:** The use value index of the most important medicinal plant species among ethnic groups

Species	Ethnic groups											
	Gedeo				Oromo				Sidama			
	Basic values			Index	Basic values			Index	Basic values			Index
	FC	UR	NU	UV	FC	UR	NU	UV	FC	UR	NU	UV
*Vachellia oerfota* (Forssk.) Kyal. & Boatwr	–	–	–	–	8	8	2	0.127	–	–	–	–
*Achyranthes aspera* L	4	6	5	0.1	7	7	5	0.111	4	7	6	0.111
*Ajuga integrifolia* Buch.-Ham. ex D.Don	–	–	–	–	–	–	–	–	10	12	5	0.19
*Albizia gummifera* (J.F.Gmel.) C.A.Sm	20	47	20	0.75	6	10	7	0.159	7	13	8	0.206
*Allium sativum* L	4	11	7	0.18	5	12	6	0.19	8	23	9	0.365
*Aloe macrocarpa* Tod	–	–	–	–	16	38	14	0.603	–	–	–	–
*Aloe vera* (L.) Burm.f	–	–	–	–	–	–	–	–	5	6	4	0.095
*Argemone mexicana* L	–	–	–	–	5	5	4	0.079	–	–	–	–
*Artemisia abyssinica* Sch.Bip. ex A.Rich	6	11	6	0.18	–	–	–	–	–	–	–	–
*Asparagus africanus* Lam	10	13	7	0.21	6	6	5	0.095	–	–	–	–
*Balanites aegyptiaca* (L.) Delile	–	–	–	–	–	–	–	–	4	7	3	0.111
*Bersama abyssinica* Fresen	4	5	4	0.08	–	–	–	–	–	–	–	–
*Calpurnia aurea* (Aiton) Benth	19	31	13	0.49	11	14	9	0.222	4	5	5	0.079
*Carica papaya* L	–	–	–	–	4	7	6	0.111	7	10	4	0.159
*Carissa spinarum* L	–	–	–	–	6	6	3	0.095	–	–	–	–
*Catha edulis* (Vahl) Forssk. ex Endl	–	–	–	–	–	–	–	–	7	9	4	0.143
*Celtis africana* Burm.f	10	18	11	0.29	–	–	–	–	–	–	–	–
*Cinnamomum verum* J.Presl	–	–	–	–	–	–	–	–	3	5	3	0.079
*Citrus limon* (L.) Osbeck	–	–	–	–	6	11	5	0.175	–	–	–	–
*Clutia abyssinica* Jaub. & Spach	–	–	–	–	6	7	6	0.111	6	6	4	0.095
*Coffea arabica* L	9	12	8	0.19	5	9	5	0.143	–	–	–	–
*Croton macrostachyus* Hochst. ex Delile	28	80	21	1.3	21	48	22	0.80	17	36	15	0.60
*Cucumis prophetarum* L	–	–	–	–	–	–	–	–	14	20	9	0.317
*Cymbopogon citratus* (DC.) Stapf	5	15	9	0.24	–	–	–	–	–	–	–	–
*Datura stramonium* L. test	–	–	–	–	6	6	3	0.095	–	–	–	–
*Dodonaea viscosa subsp. angustifolia* (L.f.) J.G.West	–	–	–	–	8	9	6	0.143	–	–	–	–
*Drynaria volkensii* Heiron	4	6	4	0.1	–	–	–	–	–	–	–	–
*Echinops kebericho* Mesfin	–	–	–	–	–	–	–	–	4	10	4	0.159
*Ehretia cymosa* Thonn	–	–	–	–	–	–	–	–	5	5	5	0.079
*Ekebergia capensis Sparrm*	5	9	5	0.14	6	14	10	0.222	15	20	8	0.317
*Ensete ventricosum* (Welw.) Cheesman	6	7	4	0.11	–	–	–	–	–	–	–	–
*Erythrina abyssinica* Lam	6	10	8	0.16	6	6	6	0.095	–	–	–	–
*Eucalyptus globulus* Labill	5	7	5	0.11	9	16	5	0.254	9	20	11	0.317
*Galinsoga quadriradiata* Ruiz & Pav	7	9	4	0.14	–	–	–	–	–	–	–	–
*Grewia ferruginea* Hochst. ex A.Rich	5	9	8	0.14	–	–	–	–	–	–	–	–
*Hagenia abyssinica* (Bruce) J.F.Gmel	–	–	–	–	5	7	5	0.111	–	–	–	–
*Justicia schimperiana* (Hochst. ex Nees) T.Anderson	6	8	5	0.13	9	11	4	0.175	8	9	5	0.143
*Kalanchoe petitiana* A.Rich	–	–	–	–	–	–	–	–	6	7	5	0.111
*Lactuca inermis* Forssk	4	9	3	0.14	–	–	–	–	–	–	–	–
*Lagenaria siceraria* (Molina) Standl	3	5	4	0.08	–	–	–	–	5	6	3	0.095
*Linum usitatissimum* L	–	–	–	–	–	–	–	–	4	8	5	0.127
*Maesa lanceolata* Forssk	7	9	7	0.14	–	–	–	–	–	–	–	–
*Melia azedarach* L	3	6	6	0.1	11	16	11	0.254	8	15	9	0.238
*Millettia ferruginea* (Hochst.) Hochst. ex Baker	6	12	10	0.19	–	–	–	–	–	–	–	–
*Momordica boivinii* Baill	–	–	–	–	–	–	–	–	7	7	6	0.111
*Moringa stenopetala* (Baker f.) Cufod	4	9	5	0.14	13	25	14	0.397	3	5	2	0.079
*Nigella sativa* L	3	7	7	0.11	7	12	10	0.19	–	–	–	–
*Ocimum jamesii* Sebald	–	–	–	–	–	–	–	–	5	5	1	0.079
*Ocimum lamiifolium* Hochst. ex Benth	3	6	6	0.1	–	–	–	–	–	–	–	–
*Ocimum gratissimum* L	7	15	6	0.24	8	14	6	0.222	–	–	–	–
*Olea europaea subsp. cuspidata* (Wall. & G.Don) Cif	9	11	6	0.18	11	25	18	0.397	–	–	–	–
*Olinia rochetiana* A.Juss	–	–	–	–	12	13	8	0.206	–	–	–	–
*Phytolacca dodecandra* L'Hér	3	5	5	0.08	–	–	–	–	9	14	7	0.222
*Coleus igniarius* Schweinf	–	–	–	–	–	–	–	–	8	13	6	0.206
*Afrocarpus falcatus* (Thunb.) C.N.Page	16	26	10	0.41	–	–	–	–	–	–	–	–
*Psidium guajava* L	–	–	–	–	3	6	4	0.095	–	–	–	–
*Psydrax schimperianus* (A.Rich.) Bridson	–	–	–	–	5	7	5	0.111	–	–	–	–
*Searsia glutinosa* (Hochst. ex A.Rich.) Moffett	–	–	–	–	–	–	–	–	12	13	3	0.206
*Ricinus communis* L	5	9	4	0.14	–	–	–	–	3	7	5	0.111
*Rotheca myricoides* (Hochst.) Steane & Mabb	–	–	–	–	–	–	–	–	11	16	7	0.254
*Rubus steudneri* Schweinf	–	–	–	–	4	8	8	0.127	–	–	–	–
*Ruta chalepensis* L	15	28	17	0.44	9	19	13	0.302	14	21	8	0.333
*Schinus molle* L	–	–	–	–	4	6	3	0.095	–	–	–	–
*Searsia natalensis* (Bernh. ex Krauss) F.A.Barkley	–	–	–	–	–	–	–	–	7	7	1	0.111
*Sida schimperiana* Hochst. ex A.Rich	4	6	6	0.1	–	–	–	–	–	–	–	–
*Solanecio gigas* (Vatke) C.Jeffrey	13	20	10	0.32	–	–	–	–	–	–	–	–
*Solanum incanum* L	–	–	–	–	5	9	3	0.143	6	6	3	0.095
*Solanum marginatum* L.f	–	–	–	–	5	7	6	0.111	–	–	–	–
*Stephania abyssinica* (Quart.-Dill. & A.Rich.) Walp	–	–	–	–	–	–	–	–	6	6	2	0.095
*Syzygium guineense* (Willd.) DC	4	6	5	0.1	5	6	5	0.095	4	6	4	0.095
*Vepris nobilis* (Delile) Mziray	–	–	–	–	4	6	5	0.095	–	–	–	–
*Trigonella foenum-graecum* L	4	12	10	0.19	–	–	–	–	–	–	–	–
*Urtica simensis* Hochst. ex A.Rich	–	–	–	–	–	–	–	–	6	6	2	0.095
*Gymnanthemum amygdalinum* (Delile) Sch.Bip	11	17	8	0.27	21	34	15	0.54	13	25	6	0.397
*Vicia faba* L	6	6	1	0.1	–	–	–	–	10	10	1	0.159
*Withania somnifera* (L.) Dunal	–	–	–	–	5	8	5	0.127	8	10	3	0.159
*Zingiber officinale* Roscoe	3	7	5	0.11	8	16	11	0.254	12	46	13	0.70
*Ziziphus spina-christi* (L.) Willd	–	–	–	–	4	10	8	0.159	–	–	–	–

N.B: Broken lines indicate the absence of a citation for the indicated species in the study area

### Informant consensus factor

Based on disease characteristics and treatment resemblances, fourteen (14) disease categories were identified from the 100 human ailments reported in the study areas (Table 3). Among these, the categories with the highest average ICF values among ethnic groups were circulatory system disorders (0.68), followed by febrile illness, reproductive organ disorders**,** and bad/evil spirit-related complications (0.66 each) across the studied ethnic groups (Table 6). In comparison, the highest plant use citation was noted for digestive system disorders, which are 100, 102, and 117 in the Sidama, Oromo, and Gedeo ethnic groups, respectively, followed by febrile illness (115, 94, and 87) in the Sidama, Oromo, and Gedeo ethnic groups (Table 6).

**Table 6:** A detailed informant consensus factor of the three ethnic groups (Sidama (S), Gedeo (G), and Oromo (O))

Category	Ailment	Species	Use report	No of species	ICF	Ethnic group
Deep sores and Cancer-like ailments	Deep sores and Cancer-like ailments	*Asparagus africanus* Lam., *Calpurnia aurea* (Aiton) Benth., *Coffea arabica* L., *Croton macrostachyus* Hochst. ex Delile, *Olea europaea subsp. cuspidata* (Wall. & G.Don) Cif., *Afrocarpus falcatus* (Thunb.) C.N.Page, *Ruta chalepensis* L	9	7	0.25	G
Deep sores and Cancer-like ailments	Deep sores and Cancer-like ailments	*Aloe macrocarpa* Tod., *Croton macrostachyus* Hochst. ex Delile, *Olea europaea subsp. cuspidata* (Wall. & G.Don) Cif	4	3	0.33	O
Deep sores and Cancer-like ailments	Deep sores and Cancer-like ailments	*Albizia gummifera* (J.F.Gmel.) C.A.Sm., *Asparagus africanus* Lam., *Calpurnia aurea* (Aiton) Benth., *Croton macrostachyus* Hochst. ex Delile, *Cymbopogon citratus* (DC.) Stapf, *Galinsoga quadriradiata* Ruiz & Pav., *Moringa stenopetala* (Baker f.) Cufod., *Olea europaea subsp. cuspidata* (Wall. & G.Don) Cif., *Afrocarpus falcatus* (Thunb.) C.N.Page	27	9	0.69	G
Deep sores and Cancer-like ailments	Deep sores and Cancer-like ailments	*Aloe macrocarpa* Tod., *Coffea arabica* L., *Croton macrostachyus* Hochst. ex Delile, *Melia azedarach* L., *Moringa stenopetala* (Baker f.) Cufod., *Olea europaea subsp. cuspidata* (Wall. & G.Don) Cif., *Olinia rochetiana* A.Juss., *Psidium guajava* L., *Psydrax schimperianus* (A.Rich.) Bridson	13	9	0.33	O
Deep sores and Cancer-like ailments	Deep sores and Cancer-like ailments	*Albizia gummifera* (J.F.Gmel.) C.A.Sm., *Clutia abyssinica* Jaub. & Spach*, Croton macrostachyus* Hochst. ex Delile, *Cucumis prophetarum* L., *Rotheca myricoides* (Hochst.) Steane & Mabb	11	5	0.6	S
Circulatory system	Anemia	*Lactuca inermis* Forssk., *Ruta chalepensis* L	5	2	0.75	G
Circulatory system	Anemia	*Ajuga integrifolia* Buch.-Ham. ex D.Don	4	1	1	S
Circulatory system	Blood pressure	*Allium sativum* L., *Cymbopogon citratus* (DC.) Stapf, *Moringa stenopetala* (Baker f.) Cufod., *Olea europaea subsp. cuspidata* (Wall. & G.Don) Cif., *Trigonella foenum-graecum* L	12	5	0.64	G
Circulatory system	Blood pressure	*Allium sativum* L., *Citrus limon* (L.) Osbeck, *Melia azedarach* L., *Moringa stenopetala* (Baker f.) Cufod., *Olea europaea subsp. cuspidata* (Wall. & G.Don) Cif., Psidium *guajava* L., *Psydrax schimperianus* (A.Rich.) Bridson, *Gymnanthemum amygdalinum* (Delile) Sch.Bip., *Zingiber officinale* Roscoe	26	9	0.68	O
Circulatory system	Blood pressure	*Linum usitatissimum* L., *Melia azedarach* L., *Moringa stenopetala* (Baker f.) Cufod., *Zingiber officinale* Roscoe	8	4	0.57	S
Circulatory system	Snake venom	*Gymnanthemum auriculiferum* (Hiern) Isawumi	3	1	1	G
Circulatory system	Snake venom	*Solanum incanum* L	4	1	1	O
Circulatory system	Snake venom	*Searsia natalensis* (Bernh. ex Krauss) F.A.Barkley	7	1	1	S
Dermal	Allergy	*Croton macrostachyus* Hochst. ex Delile	5	1	1	G
Dermal	Bath of mother after giving birth	*Artemisia abyssinica* Sch.Bip. ex A.Rich., *Cymbopogon citratus* (DC.) Stapf, *Ruta chalepensis* L., *Gymnanthemum auriculiferum* (Hiern) Isawumi	6	4	0.4	G
Dermal	Circumcision wound	*Calpurnia aurea* (Aiton) Benth., *Croton macrostachyus* Hochst. ex Delile, *Dodonaea viscosa subsp. angustifolia* (L.f.) J.G.West, *Olinia rochetiana* A.Juss	8	4	0.57	O
Dermal	Epilepsy	*Asparagus africanus* Lam., *Justicia schimperiana* T.Anderson., *Ruta chalepensis* L	4	3	0.33	G
Dermal	Eye infection	*Croton macrostachyus* Hochst. ex Delile, *Erythrina abyssinica* Lam	5	2	0.75	G
Dermal	Eye infection	*Croton macrostachyus* Hochst. ex Delile, *Ocimum gratissimum* L	4	2	0.67	O
Dermal	Eye infection	*Croton macrostachyus* Hochst. ex Delile	3	1	1	S
Dermal	Skin infection	*Albizia gummifera* (J.F.Gmel.) C.A.Sm., *Allium sativum* L., *Artemisia abyssinica* Sch.Bip. ex A.Rich., *Asparagus africanus* Lam., *Celtis africana* Burm.f., *Croton macrostachyus* Hochst. ex Delile, *Erythrina abyssinica* Lam., *Maesa lanceolata* Forssk., *Olea europaea subsp. cuspidata* (Wall. & G.Don) Cif., *Ricinus communis* L., *Gymnanthemum amygdalinum* (Delile) Sch.Bip	19	11	0.44	G
Dermal	Skin infection	*Calpurnia aurea* (Aiton) Benth., *Croton macrostachyus* Hochst. ex Delile, *Datura stramonium* L. test, *Olinia rochetiana* A.Juss., *Psydrax schimperianus* (A.Rich.) Bridson, *Gymnanthemum amygdalinum* (Delile) Sch.Bip., *Withania somnifera* (L.) Dunal	16	7	0.6	O
Dermal	Skin infection	*Datura stramonium* L. test, *Eucalyptus globulus* Labill., *Coleus igniarius* Schweinf., *Rotheca myricoides* (Hochst.) Steane & Mabb., *Ruta chalepensis* L., *Gymnanthemum amygdalinum* (Delile) Sch.Bip	14	6	0.62	S
Dermal	Swellings	*Albizia gummifera* (J.F.Gmel.) C.A.Sm., *Asparagus africanus* Lam., *Calpurnia aurea* (Aiton) Benth., *Coffea arabica* L., *Ekebergia capensis* Sparrm., *Ensete ventricosum* (Welw.) Cheesman, *Galinsoga quadriradiata* Ruiz & Pav., *Ricinus communis* L., *Ruta chalepensis* L., *Solanecio gigas* (Vatke) C.Jeffrey	29	10	0.68	G
Dermal	Swellings	*Olea europaea subsp. cuspidata* (Wall. & G.Don) Cif., *Ricinus communis* L	5	2	0.75	O
Dermal	Swellings	*Clutia abyssinica* Jaub. & Spach*, Phytolacca dodecandra* L'Hér., *Ricinus communis* L	5	3	0.5	S
Dermal	Tetanus	*Croton macrostachyus* Hochst. ex Delile	3	1	1	S
Dermal	Wound	*Calpurnia aurea* (Aiton) Benth., *Celtis africana* Burm.f., *Coffea arabica* L., *Croton macrostachyus* Hochst. ex Delile, *Afrocarpus falcatus* (Thunb.) C.N.Page, *Ricinus communis* L	10	6	0.44	G
Dermal	Wound	*Aloe macrocarpa* Tod., *Calpurnia aurea* (Aiton) Benth., *Coffea arabica* L., *Croton macrostachyus* Hochst. ex Delile*, Olea europaea subsp. cuspidata* (Wall. & G.Don) Cif., *Olinia rochetiana* A.Juss., *Psydrax schimperianus* (A.Rich.) Bridson, *Zingiber officinale* Roscoe	13	8	0.42	O
Dermal	Wound	*Croton macrostachyus* Hochst. ex Delile*, Coleus igniarius* Schweinf, *Ricinus communis* L., *Zingiber officinale* Roscoe	12	4	0.73	S
Digestive system	Amoeba	*Albizia gummifera* (J.F.Gmel.) C.A.Sm., *Calpurnia aurea* (Aiton) Benth., *Croton macrostachyus* Hochst. ex Delile*, Ekebergia capensis* Sparrm., *Ensete ventricosum* (Welw.) Cheesman, *Maesa lanceolata* Forssk., *Ocimum gratissimum* L., *Afrocarpus falcatus* (Thunb.) C.N.Page, *Solanecio gigas* (Vatke) C.Jeffrey, *Gymnanthemum amygdalinum* (Delile) Sch.Bip	23	10	0.59	G
Digestive system	Amoeba	*Calpurnia aurea* (Aiton) Benth., *Citrus limon* (L.) Osbeck, *Croton macrostachyus* Hochst. ex Delile*, Hagenia abyssinica* (Bruce) J.F.Gmel., *Gymnanthemum amygdalinum* (Delile) Sch.Bip., *Zingiber officinale* Roscoe	11	6	0.5	O
Digestive system	Amoeba	*Albizia gummifera* (J.F.Gmel.) C.A.Sm., *Balanites aegyptiaca* (L.) Delile, *Catha edulis* (Vahl) Forssk. ex Endl., *Croton macrostachyus* Hochst. ex Delile*, Cucumis prophetarum* L., *Ekebergia capensis* Sparrm., *Justicia schimperiana* (Hochst. ex Nees) T.Anderson, *Phytolacca dodecandra* L'Hér., *Coleus igniarius* Schweinf., *Rotheca myricoides* (Hochst.) Steane & Mabb., *Gymnanthemum amygdalinum* (Delile) Sch.Bip	35	11	0.71	S
Digestive system	Diarrhea	*Brucea antidysenterica* J.F.Mill., *Celtis africana* Burm.f., *Croton macrostachyus* Hochst. ex Delile*, Ekebergia capensis* Sparrm., *Solanecio gigas* (Vatke) C.Jeffrey, *Gymnanthemum amygdalinum* (Delile) Sch.Bip	11	6	0.5	G
Digestive system	Diarrhea	*Aloe macrocarpa* Tod., *Croton macrostachyus* Hochst. ex Delile*, Hagenia abyssinica* (Bruce) J.F.Gmel., *Melia azedarach* L., *Moringa stenopetala* (Baker f.) Cufod., *Solanum incanum* L., *Gymnanthemum amygdalinum* (Delile) Sch.Bip	12	7	0.45	O
Digestive system	Diarrhea	*Balanites aegyptiaca* (L.) Delile, *Clutia abyssinica* Jaub. & Spach*, Croton macrostachyus* Hochst. ex Delile*, Cucumis prophetarum* L., *Melia azedarach* L., *Rotheca myricoides* (Hochst.) Steane & Mabb., *Ruta chalepensis* L., *Gymnanthemum amygdalinum* (Delile) Sch.Bip	14	8	0.46	S
Digestive system	Gastric diseases	*Ensete ventricosum* (Welw.) Cheesman, *Maesa lanceolata* Forssk., *Solanecio gigas* (Vatke) C.Jeffrey, *Trigonella foenum-graecum* L., *Vicia faba* L	11	5	0.6	G
Digestive system	Gastric diseases	*Coffea arabica* L., *Dodonaea viscosa subsp. angustifolia* (L.f.) J.G.West*, Moringa stenopetala* (Baker f.) Cufod., *Gymnanthemum amygdalinum* (Delile) Sch.Bip., *Zingiber officinale* Roscoe	6	5	0.2	O
Digestive system	Gastric diseases	*Carica papaya* L., *Linum usitatissimum* L., *Melia azedarach* L., *Saccharum officinarum* L., *Vicia faba* L	19	5	0.78	S
Digestive system	Giardia	*Croton macrostachyus* Hochst. ex Delile*, Ruta chalepensis* L	3	2	0.5	O
Digestive system	Intestinal worms	*Albizia gummifera* (J.F.Gmel.) C.A.Sm., *Calpurnia aurea* (Aiton) Benth., *Celtis africana* Burm.f., *Croton macrostachyus* Hochst. ex Delile*, Gymnanthemum amygdalinum* (Delile) Sch.Bip	10	5	0.56	G
Digestive system	Intestinal worms	*Aloe macrocarpa* Tod., *Croton macrostachyus* Hochst. ex Delile*, Melia azedarach* L., *Moringa stenopetala* (Baker f.) Cufod., *Olea europaea subsp. cuspidata* (Wall. & G.Don) Cif., *Psidium guajava* L., *Gymnanthemum amygdalinum* (Delile) Sch.Bip	11	7	0.4	O
Digestive system	Intestinal worms	*Carica papaya* L., *Phytolacca dodecandra* L'Hér	3	2	0.5	S
Digestive system	Jaundice	*Asparagus africanus* Lam., *Calpurnia aurea* (Aiton) Benth., *Celtis africana* Burm.f., *Coffea arabica* L., *Justicia schimperiana* (Hochst. ex Nees) T.Anderson, *Maesa lanceolata* Forssk., *Moringa stenopetala* (Baker f.) Cufod., *Afrocarpus falcatus* (Thunb.) C.N.Page, *Ruta chalepensis* L., *Solanecio gigas* (Vatke) C.Jeffrey	22	10	0.57	G
Digestive system	Jaundice	*Aloe macrocarpa* Tod., *Croton macrostachyus* Hochst. ex Delile*, Justicia schimperiana* T.Anderson., *Melia azedarach* L., *Moringa stenopetala* (Baker f.) Cufod., *Olea europaea subsp. cuspidata* (Wall. & G.Don) Cif., *Ruta chalepensis* L., *Schinus molle* L., *Gymnanthemum amygdalinum* (Delile) Sch.Bip	31	9	0.73	O
Digestive system	Jaundice	*Albizia gummifera* (J.F.Gmel.) C.A.Sm., *Croton macrostachyus* Hochst. ex Delile*, Cucumis prophetarum* L., *Ekebergia capensis* Sparrm., *Lagenaria siceraria* (Molina) Standl., *Ricinus communis* L., *Rotheca myricoides* (Hochst.) Steane & Mabb., *Stephania abyssinica* (Quart.-Dill. & A.Rich.) Walp	12	8	0.36	S
Digestive system	Stomachache	*Albizia gummifera* (J.F.Gmel.) C.A.Sm., *Allium sativum* L., *Brucea antidysenterica* J.F.Mill., *Celtis africana* Burm.f, *Croton macrostachyus* Hochst. ex Delile*, Cymbopogon citratus* (DC.) Stapf, *Ekebergia capensis* Sparrm., *Lactuca inermis* Forssk., *Ocimum gratissimum* L., *Ruta chalepensis* L., *Trigonella foenum-graecum* L., *Gymnanthemum amygdalinum* (Delile) Sch.Bip	31	12	0.63	G
Digestive system	Stomachache	*Aloe macrocarpa* Tod., *Calpurnia aurea* (Aiton) Benth., Citrus limon (L.) Burm.F., *Rotheca myricoides* (Hochst.) Steane & Mabb*, Croton macrostachyus* Hochst. ex Delile*, Dodonaea viscosa subsp. angustifolia* (L.f.) J.G.West*, Melia azedarach* L., *Ocimum gratissimum* L., *Olinia rochetiana* A.Juss., *Ruta chalepensis* L., *Gymnanthemum amygdalinum* (Delile) Sch.Bip	21	11	0.5	O
Digestive system	Stomachache	*Ajuga integrifolia* Buch.-Ham. ex D.Don, *Albizia gummifera* (J.F.Gmel.) C.A.Sm., *Balanites aegyptiaca* (L.) Delile*, Ekebergia capensis* Sparrm., *Justicia schimperiana* (Hochst. ex Nees) T.Anderson, *Melia azedarach* L., *Phytolacca dodecandra* L'Hér., *Gymnanthemum amygdalinum* (Delile) Sch.Bip	14	8	0.46	S
Digestive system	Tapeworm	*Hagenia* abyssinica (Brace) J.F.Gmel	3	1	1	O
Digestive system	Vomiting	*Cymbopogon citratus* (DC.) Stapf, Ruta chalepensis L	3	2	0.5	G
Digestive system	Vomiting	*Ocimum gratissimum* L., *Ruta chalepensis* L., *Gymnanthemum amygdalinum* (Delile) Sch.Bip	4	3	0.33	O
Digestive system	Weight loss	*Lactuca inermis* Forssk., *Trigonella foenum-graecum* L	6	2	0.8	G
Digestive system	Weight loss	*Ajuga integrifolia* Buch.-Ham. ex D.Don*, Linum usitatissimum* L	3	2	0.5	S
Febrile illness	Dizziness	*Albizia gummifera* (J.F.Gmel.) C.A.Sm., *Croton macrostachyus* Hochst. ex Delile*,*	18	2	0.94	G
Febrile illness	Dizziness	*Croton macrostachyus* Hochst. ex Delile	4	1	1	O
Febrile illness	Dizziness	*Albizia gummifera* (J.F.Gmel.) C.A.Sm., *Croton macrostachyus* Hochst. ex Delile*,*	8	2	0.86	S
Febrile illness	Sudden sickness (Dingetegna) in the local language	*Albizia gummifera* (J.F.Gmel.) C.A.Sm., *Artemisia abyssinica* Sch.Bip. ex A.Rich., *Calpurnia aurea* (Aiton) Benth., *Croton macrostachyus* Hochst. ex Delile*, Ekebergia capensis* Sparrm., *Ocimum gratissimum* L*.*, *Ruta chalepensis* L	23	7	0.73	G
Febrile illness	Sudden sickness (Dingetegna) in the local language	*Allium sativum* L., *Carissa spinarum* L., *Croton macrostachyus* Hochst. ex Delile*, Hagenia* abyssinica (Brace) J.F.Gmel., *Ocimum gratissimum* L., *Psydrax schimperianus* (A.Rich.) Bridson, *Ruta chalepensis* L., *Gymnanthemum amygdalinum* (Delile) Sch.Bip., *Withania somnifera* (L.) Dunal, *Zingiber officinale* Roscoe	27	10	0.65	O
Febrile illness	Sudden sickness (Dingetegna) in the local language	*Allium sativum* L., *Croton macrostachyus* Hochst. ex Delile*, Echinops kebericho* Mesfin, *Ocimum jamesii* Sebald, *Coleus igniarius* Schweinf., *Ruta chalepensis* L	22	6	0.76	S
Febrile illness	Fever	*Albizia gummifera* (J.F.Gmel.) C.A.Sm., *Erythrina abyssinica* Lam., *Ocimum gratissimum* L., *Solanecio gigas* (Vatke) C.Jeffrey	6	4	0.4	G
Febrile illness	Fever	*Allium sativum* L., *Aloe macrocarpa* Tod., *Calpurnia aurea* (Aiton) Benth., *Citrus limon* (L.) Osbeck, *Melia azedarach* L., *Ocimum gratissimum* L., *Zingiber officinale* Roscoe	11	7	0.4	O
Febrile illness	Fever	*Allium sativum* L., *Carica papaya* L., *Ocimum gratissimum* L., *Ekebergia capensis* Sparrm., *Eucalyptus globulus* Labill., *Zingiber officinale* Roscoe	10	6	0.44	S
Febrile illness	Headache	*Artemisia abyssinica* Sch.Bip. ex A.Rich., *Calpurnia aurea* (Aiton) Benth., *Celtis africana* Burm.f, *Ruta chalepensis* L	4	4	0	G
Febrile illness	Headache	*Calpurnia aurea* (Aiton) Benth., *Carissa spinarum* L., *Dodonaea viscosa subsp. angustifolia* (L.f.) J.G.West*, Eucalyptus globulus* Labill	5	4	0.25	O
Febrile illness	Headache	*Allium sativum* L., *Echinops kebericho* Mesfin, *Eucalyptus globulus* Labill., *Zingiber officinale* Roscoe	8	4	0.57	S
Febrile illness	Malaria	*Albizia gummifera* (J.F.Gmel.) C.A.Sm., *Croton macrostachyus* Hochst. ex Delile*, Erythrina abyssinica* Lam., *Justicia schimperiana* (Hochst. ex Nees) T.Anderson, *Ocimum gratissimum* L., *Afrocarpus falcatus* (Thunb.) C.N.Page, *Ruta chalepensis* L., *Solanecio gigas* (Vatke) C.Jeffrey, *Gymnanthemum amygdalinum* (Delile) Sch.Bip	18	9	0.53	G
Febrile illness	Malaria	*Aloe macrocarpa* Tod., *Croton macrostachyus* Hochst. ex Delile*, Moringa stenopetala* (Baker f.) Cufod., *Ocimum gratissimum* L., *Olea europaea subsp. cuspidata* (Wall. & G.Don) Cif., *Ruta chalepensis* L., *Gymnanthemum amygdalinum* (Delile) Sch.Bip	21	7	0.7	O
Febrile illness	Malaria	*Ajuga integrifolia* Buch.-Ham. ex D.Don, *Allium sativum* L., *Carica papaya* L., *Melia azedarach* L., *Gymnanthemum amygdalinum* (Delile) Sch.Bip., *Zingiber officinale* Roscoe	30	6	0.83	S
Febrile illness	Pain relief	*Aloe macrocarpa* Tod., *Moringa stenopetala* (Baker f.) Cufod., *Olea europaea subsp. cuspidata* (Wall. & G.Don) Cif.,	3	3	0	O
Febrile illness	Pain relief	*Ajuga integrifolia* Buch.-Ham. ex D.Don*, Eucalyptus globulus* Labill., *Melia azedarach* L	3	3	0	S
Febrile illness	Tonsillitis	*Galinsoga quadriradiata* Ruiz & Pav., *Ricinus communis* L	8	2	0.86	G
Febrile illness	Tonsillitis	*Allium sativum* L., *Schinus molle* L., *Zingiber officinale* Roscoe	5	3	0.5	O
Febrile illness	Tonsillitis	*Allium sativum* L., *Ricinus communis* L., *Zingiber officinale* Roscoe	16	3	0.87	S
Febrile illness	Typhoid	*Albizia gummifera* (J.F.Gmel.) C.A.Sm., *Allium sativum* L., *Croton macrostachyus* Hochst. ex Delile*, Afrocarpus falcatus* (Thunb.) C.N.Page, *Ruta chalepensis* L., *Gymnanthemum amygdalinum* (Delile) Sch.Bip	14	6	0.62	G
Febrile illness	Typhoid	*Allium sativum* L., *Aloe macrocarpa* Tod., *Calpurnia aurea* (Aiton) Benth., *Croton macrostachyus* Hochst. ex Delile*, Melia azedarach* L., *Moringa stenopetala* (Baker f.) Cufod., *Psidium guajava* L., *Ruta chalepensis* L., *Zingiber officinale* Roscoe	21	9	0.6	O
Febrile illness	Typhoid	*Allium sativum* L., *Ekebergia capensis* Sparrm., *Ruta chalepensis* L., *Zingiber officinale* Roscoe	21	4	0.85	S
Genitourinary system	Kidney infection	*Cymbopogon citratus* (DC.) Stapf, *Moringa stenopetala* (Baker f.) Cufod., *Ocimum gratissimum* L	3	3	0	G
Genitourinary system	Kidney infection	*Coffea arabica* L., *Moringa stenopetala* (Baker f.) Cufod., *Olea europaea subsp. cuspidata* (Wall. & G.Don) Cif	4	3	0.33	O
Gland	Glandular	*Albizia gummifera* (J.F.Gmel.) C.A.Sm., *Calpurnia aurea* (Aiton) Benth., *Celtis africana* Burm.f, *Croton macrostachyus* Hochst. ex Delile*, Justicia schimperiana* (Hochst. ex Nees) T.Anderson, *Moringa stenopetala* (Baker f.) Cufod., *Afrocarpus falcatus* (Thunb.) C.N.Page, *Ruta chalepensis* L., *Solanecio gigas* (Vatke) C.Jeffrey	16	9	0.47	G
Gland	Glandular	*Justicia schimperiana* (Hochst. ex Nees) T.Anderson, *Melia azedarach* L., *Moringa stenopetala* (Baker f.) Cufod., *Olinia rochetiana* A.Juss	6	4	0.4	O
Gland	Glandular	*Croton macrostachyus* Hochst. ex Delile*, Cucumis prophetarum* L., *Lagenaria siceraria* (Molina) Standl., *Moringa stenopetala* (Baker f.) Cufod., *Searsia glutinosa* (Hochst. ex A.Rich.) Moffett, *Rotheca myricoides* (Hochst.) Steane & Mabb., *Stephania abyssinica* (Quart.-Dill. & A.Rich.) Walp	24	7	0.74	S
Gland	Goiter	*Albizia gummifera* (J.F.Gmel.) C.A.Sm., *Ekebergia capensis* Sparrm., *Ruta chalepensis* L., *Zingiber officinale* Roscoe	6	4	0.4	S
Lightning	Lightning	*Croton macrostachyus* Hochst. ex Delile*, Ensete ventricosum* (Welw.) Cheesman	6	2	0.8	G
Mental	Depression	*Catha edulis* (Vahl) Forssk. ex Endl., *Melia azedarach* L	3	2	0.5	S
Mental	Rabies	*Rotheca myricoides* (Hochst.) Steane & Mabb, *Datura stramonium* L. test*, Gymnanthemum amygdalinum* (Delile) Sch.Bip	5	3	0.5	O
Mental	Rabies	*Antiaris toxicaria* (J.F.Gmel.) Lesch., *Justicia schimperiana* (Hochst. ex Nees) T.Anderson	5	2	0.75	S
Musculoskeletal	Cancer-like ailments	*Calpurnia aurea* (Aiton) Benth., *Croton macrostachyus* Hochst. ex Delile	3	2	0.5	G
Periodontal	Toothache	*Albizia gummifera* (J.F.Gmel.) C.A.Sm., *Coffea arabica* L., *Galinsoga quadriradiata* Ruiz & Pav., *Olea europaea subsp. cuspidata* (Wall. & G.Don) Cif., *Afrocarpus falcatus* (Thunb.) C.N.Page	7	5	0.33	G
Periodontal	Toothache	*Calpurnia aurea* (Aiton) Benth., *Datura stramonium* L. test*, Melia azedarach* L., *Olea europaea subsp. cuspidata* (Wall. & G.Don) Cif., *Olinia rochetiana* A.Juss	7	5	0.33	O
Reproductive	Abortion	*Cymbopogon citratus* (DC.) Stapf	3	1	1	G
Reproductive	Abortion	*Phytolacca dodecandra* L'Hér	4	1	1	S
Reproductive	Gonorrhea	*Albizia gummifera* (J.F.Gmel.) C.A.Sm., *Croton macrostachyus* Hochst. ex Delile*, Cymbopogon citratus* (DC.) Stapf, *Maesa lanceolata* Forssk., *Afrocarpus falcatus* (Thunb.) C.N.Page, *Ruta chalepensis* L	24	6	0.78	G
Reproductive	Gonorrhea	*Aloe macrocarpa* Tod., *Croton macrostachyus* Hochst. ex Delile*, Hagenia abyssinica* (Bruce) J.F.Gmel., *Ruta chalepensis* L., *Gymnanthemum amygdalinum* (Delile) Sch.Bip	11	5	0.6	O
Reproductive	Gonorrhea	*Allium sativum* L., *Brucea antidysenterica* J.F.Mill., *Catha edulis* (Vahl) Forssk. ex Endl., *Croton macrostachyus* Hochst. ex Delile*, Ekebergia capensis* Sparrm., *Justicia schimperiana* T.Anderson, *Phytolacca dodecandra* L'Hér., *Afrocarpus falcatus* (Thunb.) C.N.Page, *Ruta chalepensis* L	27	9	0.69	S
Reproductive	Menstruation cycle disorder	*Albizia gummifera* (J.F.Gmel.) C.A.Sm., *Croton macrostachyus* Hochst. ex Delile*, Ruta chalepensis* L., *Trigonella foenum-graecum* L	5	4	0.25	G
Respiratory	Asthma	*Allium sativum* L., *Celtis africana* Burm.f, *Ruta chalepensis* L	3	3	0	G
Respiratory	Asthma	*Croton macrostachyus* Hochst. ex Delile*, Eucalyptus globulus* Labill., *Olea europaea subsp. cuspidata* (Wall. & G.Don) Cif., *Withania somnifera* (L.) Dunal	11	4	0.7	O
Respiratory	Asthma	*Eucalyptus globulus* Labill., *Withania somnifera* (L.) Dunal, *Zingiber officinale* Roscoe	8	3	0.71	S
Respiratory	Common cold	*Allium sativum* L	3	1	1	G
Respiratory	Common cold	*Allium sativum* L., *Citrus limon* (L.) Osbeck*, Eucalyptus globulus* Labill., *Zingiber officinale* Roscoe	14	4	0.77	O
Respiratory	Common cold	*Allium sativum* L., *Echinops kebericho* Mesfin*, Eucalyptus globulus* Labill., *Zingiber officinale* Roscoe	27	4	0.88	S
Respiratory	Cough	*Albizia gummifera* (J.F.Gmel.) C.A.Sm., *Erythrina abyssinica* Lam., *Maesa lanceolata* Forssk., *Trigonella foenum-graecum* L	7	4	0.5	G
Respiratory	Lung infection	*Albizia gummifera* (J.F.Gmel.) C.A.Sm., *Asparagus africanus* Lam*., Celtis africana* Burm.f, *Erythrina abyssinica* Lam., *Solanecio gigas* (Vatke) C.Jeffrey, *Trigonella foenum-graecum* L	6	6	0	G
Respiratory	Lung infection	*Aloe macrocarpa* Tod., *Croton macrostachyus* Hochst. ex Delile*, Dodonaea viscosa subsp. angustifolia* (L.f.) J.G.West, *Moringa stenopetala* (Baker f.) Cufod., *Ricinus communis* L., *Gymnanthemum amygdalinum* (Delile) Sch.Bip	6	6	0	O
Respiratory	Lung infection	*Albizia gummifera* (J.F.Gmel.) C.A.Sm., *Croton macrostachyus* Hochst. ex Delile*, Cucumis prophetarum* L., *Lagenaria siceraria* (Molina) Standl., *Searsia glutinosa* (Hochst. ex A.Rich.) Moffett*, Ricinus communis* L., *Rotheca myricoides* (Hochst.) Steane & Mabb	17	7	0.62	S
Respiratory	Nasal bleeding	*Ruta chalepensis* L., *Solanecio gigas* (Vatke) C.Jeffrey	3	2	0.5	G
Respiratory	Nasal bleeding	*Eucalyptus globulus* Labill., *Ruta chalepensis* L., *Schinus molle* L., *Solanum incanum* L	7	4	0.5	O
Respiratory	Sneezing	*Coffea arabica* L.,	3	1	1	O
Respiratory	Tuberculosis	*Albizia gummifera* (J.F.Gmel.) C.A.Sm., *Erythrina abyssinica* Lam., *Trigonella foenum-graecum* L	3	3	0	G
Respiratory	Tuberculosis	*Olea europaea subsp. cuspidata* (Wall. & G.Don) Cif., *Olinia rochetiana* A.Juss., *Ruta chalepensis* L	3	3	0	O
Respiratory	Tuberculosis	*Ekebergia capensis* Sparrm., *Ruta chalepensis* L	4	2	0.67	S
Bad/evil spirit	Bad/evil spirit	*Albizia gummifera* (J.F.Gmel.) C.A.Sm., *Artemisia abyssinica* Sch. Bip. ex A. Rich., *Calpurnia aurea* (Aiton) Benth., *Croton macrostachyus* Hochst. ex Delile*, Withania somnifera* (L.) Dunal	11	5	0.6	G
Bad/evil spirit	Bad/evil spirit	*Vachellia oerfota* (Forssk.) Kyal. & Boatwr., *Carissa spinarum* L., *Rotheca myricoides* (Hochst.) Steane & Mabb*, Olea europaea subsp. cuspidata* (Wall. & G.Don) Cif., *Withania somnifera* (L.) Dunal	17	5	0.75	O
Bad/evil spirit	Bad/evil spirit	*Catha edulis* (Vahl) Forssk. ex Endl., *Clutia abyssinica* Jaub. & Spach*, Croton macrostachyus* Hochst. ex Delile*, Eucalyptus globulus* Labill., *Coleus igniarius* Schweinf., *Searsia glutinosa* (Hochst. ex A.Rich.) Moffett*, Ruta chalepensis* L., *Urtica simensis* Hochst. ex A.Rich., *Withania somnifera* (L.) Dunal	23	9	0.64	S

### Fidelity level (FLs), relative popularity level (RPL), and rank-order priority (ROP)

The relative healing potential of medicinal plants is calculated for plants at least cited by three or more informants against particular ailments, and the FLs, RPL, and ROP values ranged from 50 to 100%, 0.5 to 1, and 24 to 100%, respectively (Table 7). Twenty-six plant species were identified as the most preferred plants (ROP > 50%) across ethnic groups (Table 7). *Lactuca inermis* Forssk., *Moringa stenopetala* (Baker f.) Cufod., and *Withania somnifera* (L.) scored the highest FLs and ROP values in the Gedeo ethnic group, whereas *Allium sativum* L., *Citrus limon* (L.) Osbeck, *Ricinus communis* L., and *Schinus molle* L., in the Oromo ethnic group, and *Antiaris toxicaria* (J.F.Gmel.) Lesch., *Brucea antidysenterica* J.F.Mill., *Echinops kebericho* Mesfin, *Ocimum jamesii* Sebald, *Afrocarpus falcatus* (Thunb.) C.N.Page, *Searsia natalensis* (Bernh. ex Krauss) F.A.Barkley, and *Ricinus communis* L. in the Sidama ethnic group (Table 7).

**Table 7:** The most curative medicinal plant species with their FL, RPL, and ROP values among ethnic groups

Ethnic groups	Species name	Ailment treated	FL (%)	RPL	ROP
Gedeo	*Achyranthes aspera* L	Jaundice	50	0.5	25
	*Allium sativum* L	Stomachache	50	0.5	25
	*Allium sativum* L	Common cold	75	0.75	56
	*Artemisia abyssinica* Sch.Bip. ex A.Rich	Bad/evil spirit	66.67	0.67	45
	*Brucea antidysenterica* J.F.Mill	Diarrhea	75	0.75	56
	*Celtis africana* Burm.f	Stomachache	50	0.5	25
	*Commelina benghalensis* L	Skin infection	66.67	0.67	45
	*Clutia abyssinica* Jaub. & Spach	Deep sores /cancer-like ailments	50	0.5	25
	*Cymbopogon citratus* (DC.) Stapf	Blood pressure	80	0.8	64
	*Cymbopogon citratus* (DC.) Stapf	Abortion	60	0.6	36
	*Datura stramonium* L. test	Rabies	66.67	0.67	45
	*Drynaria volkensii* Heiron	Swellings	50	0.5	25
	*Drynaria volkensii* Heiron	Ear infection	50	0.5	25
	*Ekebergia capensis* Sparrm	Diarrhea	66.67	0.67	45
	*Ekebergia capensis* Sparrm	Stomachache	66.67	0.67	45
	*Ensete ventricosum* (Welw.) Cheesman	Lightning	66.67	0.67	45
	*Erythrina abyssinica* Lam	Cough		0.5	25
	*Galinsoga quadriradiata* Ruiz & Pav	Tonsillitis	85.71	0.86	74
	*Grewia ferruginea* Hochst. ex A.Rich	Headache	66.67	0.67	45
	*Hyparrhenia rufa* (Nees) Stapf	Swellings	66.67	0.67	45
	*Justicia schimperiana* (Hochst. ex Nees) T.Anderson	Jaundice	50	0.5	25
	*Lactuca inermis* Forssk	Anemia	100	1	100
	*Lactuca inermis* Forssk	Stomachache	50	0.5	25
	*Lagenaria siceraria* (Molina) Standl	Amoeba	66.67	0.67	45
	*Moringa stenopetala* (Baker f.) Cufod	Blood pressure	100	1	100
	*Moringa stenopetala* (Baker f.) Cufod	Glandular	50	0.5	25
	*Ocimum gratissimum* L	Febrile illness	71.43	0.71	51
	*Coleus igniarius* Schweinf	Stomachache	66.67	0.67	45
	*Ricinus communis* L	Swellings	80	0.8	64
	*Syzygium guineense* (Willd.) DC	Pain relief	66.67	0.67	45
	*Solanecio gigas* (Vatke) C.Jeffrey	Jaundice	53.85	0.54	29
	*Gymnanthemum auriculiferum* (Hiern) Isawumi	Snake venom	66.67	0.67	45
	*Withania somnifera* (L.) Dunal	Bad/evil spirit	100	1	100
	*Zingiber officinale* Roscoe	Tonsillitis	66.67	0.67	45
Oromo	*Vachellia oerfota* (Forssk.) Kyal. & Boatwr	Bad/evil spirit	87.50	0.88	77
	*Allium sativum* L	Common cold	100	1	100
	*Allium sativum* L	Typhoid	60	0.4	24
	*Allium sativum* L	Tonsillitis	60	0.4	24
	*Aloe macrocarpa* Tod	Malaria	62.5	0.62	39
	*Aloe macrocarpa* Tod	Typhoid	50	0.5	25
	*Albizia gummifera* (J.F.Gmel.) C.A.Sm	Dizziness	50	0.5	25
	*Brassica carinata* A.Braun	Fever	66.67	0.67	45
	*Carissa spinarum* L	Bad/evil spirit	66.67	0.67	45
	*Citrus limon* (L.) Osbeck	Blood pressure	100	1	100
	*Rotheca myricoides* (Hochst.) Steane & Mabb	Stomachache	75	0.5	38
	*Coffea arabica* L	Sneezing	60	0.6	36
	*Ekebergia capensis* Sparrm	Skin infection	50	0.5	25
	*Erica arborea* L	Wound	66.67	0.67	45
	*Eucalyptus globulus* Labill	Asthma	77.78	0.78	61
	*Eucalyptus globulus* Labill	Common cold	53.85	0.54	29
	*Ficus sycomorus* L	Tonsillitis	66.67	0.67	45
	*Hagenia abyssinica* (Bruce) J.F.Gmel	Tapeworm	60	0.6	36
	*Justicia schimperiana* (Hochst. ex Nees) T.Anderson	Jaundice	77.78	0.78	61
	*Phytolacca dodecandra* L'Hér	Abortion	66.67	0.67	45
	*Ocimum lamiifolium* Hochst. ex Benth	Febrile illness	66.67	0.67	45
	*Ocimum gratissimum* L	Febrile illness	87.5	0.75	66
	*Searsia natalensis* (Bernh. ex Krauss) F.A.Barkley	Autism	50	05	25
	*Ricinus communis* L	Swellings	100	1	100
	*Solanum incanum* L	Nasal bleeding	80	0.8	64
	*Schinus molle* L	Jaundice	100	1	100
	*Stephania abyssinica* (Quart.-Dill. & A.Rich.) Walp	Gonorrhea	50	0.5	25
	*Withania somnifera* (L.) Dunal	Bad/evil spirit	66.67	0.6	36
	*Ziziphus spina-christi* (L.) Willd	Gonorrhea	50	0.5	25
	*Ziziphus spina-christi* (L.) Willd	Bad/evil spirit	50	0.5	25
Sidama	*Achyranthes aspera* L	Headache	50	0.5	25
	*Albizia gummifera* (J.F.Gmel.) C.A.Sm	Dizziness	57.14	0.57	33
	*Allium sativum* L	Fever	50	0.38	19
	*Allium sativum* L	Typhoid	87.5	0.75	66
	*Antiaris toxicaria* (J.F.Gmel.) Lesch	Rabies	100	1	100
	*Balanites aegyptiaca* (L.) Delile	Amoeba	75	0.75	56
	*Balanites aegyptiaca* (L.) Delile	Diarrhea	75	0.75	56
	*Brucea antidysenterica* J.F.Mill	Gonorrhea	100	1	100
	*Carica papaya* L	Malaria	100	0.86	86
	*Carissa spinarum* L	Diarrhea	66.67	0.67	45
	*Catha edulis* (Vahl) Forssk. ex Endl	Gonorrhea	71.43	0.71	51
	*Cinnamomum verum* J.Presl	Asthma	66.67	0.67	45
	*Cinnamomum verum* J.Presl	Common cold	66.67	0.67	45
	*Clutia abyssinica* Jaub. & Spach	Diarrhea	50	0.5	25
	*Echinops kebericho* Mesfin	Common cold	100	1	100
	*Echinops kebericho* Mesfin	Febrile illness	100	1	100
	*Echinops kebericho* Mesfin	Headache	75	0.75	56
	*Ekebergia capensis* Sparrm	Amoeba	60	0.6	36
	*Eucalyptus globulus* Labill	Common cold	60	0.54	29
	*Lagenaria siceraria* (Molina) Standl	Jaundice	60	0.6	36
	*Melia azedarach* L	Malaria	75	0.62	47
	*Moringa stenopetala* (Baker f.) Cufod	Glandular	66.67	0.67	45
	*Ocimum jamesii* Sebald	Febrile illness	100	1	100
	*Afrocarpus falcatus* (Thunb.) C.N.Page	Gonorrhea	100	1	100
	*Searsia natalensis* (Bernh. ex Krauss) F.A.Barkley	Glandular	83.33	0.83	69
	*Ricinus communis* L	Swellings	100	1	100
	*Ricinus communis* L	Tonsillitis	66.67	0.33	22
	*Ruta chalepensis* L	Typhoid	57.14	0.57	33
	*Searsia natalensis* (Bernh. ex Krauss) F.A.Barkley	Snake venom	100	1	100
	*Stephania abyssinica* (Quart.-Dill. & A.Rich.) Walp	Jaundice	50	0.5	25
	*Stephania abyssinica* (Quart.-Dill. & A.Rich.) Walp	Glandular	50	0.5	25
	*Syzygium guineense* (Willd.) DC	Amoeba	50	0.5	25
	*Syzygium guineense* (Willd.) DC	Bad/evil spirit	50	0.5	25
	*Taverniera abyssinica* A.Rich	Febrile illness	66.67	0.67	45
	*Urtica simensis* Hochst. ex A.Rich	Bad/evil spirit	83.33	0.83	69
	*Gymnanthemum amygdalinum* (Delile) Sch.Bip	Amoeba	61.54	0.62	38
	*Gymnanthemum amygdalinum* (Delile) Sch.Bip	Malaria	53.85	0.54	29
	*Withania somnifera* (L.) Dunal	Bad/evil spirit	75	0.75	56
	*Zingiber officinale* Roscoe	Tonsillitis	100	0.83	83
	*Zingiber officinale* Roscoe	Malaria	50	0.5	25
	*Zingiber officinale* Roscoe	Wound	50	0.5	25

## Discussion

The medicinal plant resources and their associated indigenous and local ethnobotanical knowledge documented revealed time-honored ethnobotanical knowledge practices of the three ethnic groups studied (Table 3). The plant families Fabaceae, Asteraceae, Lamiaceae, and Poaceae were the most abundant, followed by Solanaceae, Rutaceae, and Euphorbiaceae. The hypothesis by [39–42] that the dominance of these families in disease treatment may be due to their aromatic properties and abundance of essential oil is supported by our record of a higher number of relevant plant species from Fabaceae, Asteraceae, and Lamiaceae (Table 3). Of the total 189 medicinal plants collected, several studies conducted in Ethiopia and abroad reported diverse amounts of therapeutic plants, which witnessed about the relevance of several traditional medicinal plants documented in this study. For instance, Regassa et al. [28], Woldeamanuel et al. [56], Eshete and Molla [10], Mekuria and Abduro [57], Marshet and Dalle [58], Kidane et al. [13], Tefera and Kim [8], and Teka et al. [5] compiled 39, 39, 41, 67, 59, 71, 64, and 88 medicinal plant species, respectively, in their ethnobotanical investigations in different parts of Ethiopia. Tugume et al. [59], Hussain et al. [60], Faruque et al. [61], Lautenschläger et al. [62], Wiryono et al. [63] and Al-Robai et al. [64] documented 33, 12, 13, 22, 13, and 20 therapeutic plants, respectively, in Uganda, Pakistani-Afghan borders, Bangladesh, Angola, Indonesia, and Saudi Arabia.

In this study, *Croton macrostachyus* Hochst. ex Delile is reported as curing plants against deep sores and cancer-like ailments, eye infections, abrupt lightning, tetanus, lung infection, gonorrhea, dizziness, febrile illness, wounds, bad/evil spirit, diarrhea, jaundice, amoeba, glandular, giardia, abortion, intestinal worms, malaria, asthma, typhoid, skin infection, placental delay during birth, circumcision wound, stomachache, ear infection, allergy, and menstruation cycle disorder. Similarly, [5, 52] reported the efficacy of the species against wounds, blood clotting, tinea versicolor, common wart, nasal congestion, indigestion, abdominal pain, bloating, intestinal parasite, retained placenta, general malaise (Michi), headache, jaundice, malaria, rabies, swelling, allergies, eye infection, and pyoderma in their ethnobotanical investigations in the south-central and southwestern parts of the country. In another study, in the southern parts of Ethiopia [8, 10], the medicinal values of this species were revealed against toothache, swelling and forming deep openings, cold disease, gonorrhea, amoeboid, wounds, kidney infection, ringworm, stomachache, hepatitis, shivering, abnormal breathing, tuberculosis, gastritis, and goiter, and against jaundice in the northern parts of Ethiopia [13]. Besides, [65] reported the efficacy of this species against typhoid, measles, and skin diseases in Kenya, [59] against headache in Uganda, and [66] against abdominal pain in Tanzania.

This study revealed ethnomedicinal values of *Zingiber officinale* Roscoe against asthma, blood pressure, passive sexual interest, common cold, tonsillitis, typhoid, headache, malaria, wounds, fever, cough, tung infection, goiter, constipation, febrile illness, amoeba, gastric diseases, and stomachache. Correspondingly, it was also reported against tonsillitis, abdominal pain, toothache, common cold, and coughing in other studies in the south-central and southwestern parts of Ethiopia [5, 52]. In addition, against eye disease in Hawassa zuria districts [8]. Abroad [67] reported the significance of *Zingiber officinale* Roscoe against abdominal problems, laxative dyspepsia, dysentery and vomiting, coughs, bronchitis, asthma, and tuberculosis in Bangladesh; [68] reported against respiratory, digestive, and sexual disorders in India, and [64] reported against GIT disorders, respiratory disorders, CNS disorders, hematological disorders, endocrine disorders, rheumatic disorders, orthopedic disorders, immunological disorders, and antibacterial activities in Saudi Arabia. *Albizia gummifera* (J.F.Gmel.) C.A.Sm. is reported against deep sores and cancer-like ailments, goiter, toothache, dizziness, stomachache, jaundice, lung infection, amoeba, malaria, fire accident, skin infection, epilepsy, febrile illness, glandular, gonorrhea, swellings, fever, bad/evil spirit, cough, tuberculosis, menstruation cycle disorder, typhoid, and intestinal worms in this study. Correspondingly, [8] witnessed the significance of this species against swelling of the stomach and evil eye in Hawassa districts, southern Ethiopia, and [13] against febrile illness in northern Ethiopia. Other use reports indicated the novelty of this species against different ailments in the study areas.

*Aloe macrocarpa* Tod. is a novel for use against malaria, jaundice, typhoid, fever, deep sores and cancer-like ailment, lung infection, gonorrhea, pain relief, urinary organ infection, intestinal worms, wounds, stomachache, and diarrhea, because this is a new report and has not been reported before. However, [13] reported *Aloe megalacantha* Baker and *Aloe camperi* Schweinf. against dislocated bone, malaria, hemorrhoid, and eye disease; [14] reported *Aloe weloensis* Sebsebe against wounds, and malaria in northern Ethiopia, respectively; [28] reported *Aloe gilbertii* T. Reynolds ex Sebsebe and Brandham against malaria, intestinal parasites, tonsillitis, wounds, stomach pain, sudden disease, constipation, and eye problems in southern Ethiopia; and [21] reported *Aloe otallensis* Baker against blood clothing, wounds, and tuberculosis in southwestern Ethiopia. *Calpurnia aurea* (Aiton) Benth. is reported against lung infection, typhoid, intestinal worms, jaundice, bad/evil spirit, amoeba, headache, stomachache, toothache, fever, skin infection, wound, circumcision wound, febrile illness, deep sores and cancer-like ailment, swellings, glandular, and respiratory organ infection in our investigation. In addition, Eshete and Molla [10] reported this medicinal plant species against hepatitis, ear ache, and hypertension in the southern parts of the country. Likewise, [5] reported against toothache in south-central and in addition, [13] reported against eye diseases in the northern parts of the country. *Gymnanthemum amygdalinum* (Delile) Sch.Bip. is reported against amoeba, malaria, skin infection, stomachache, diarrhea, head skin infection, gonorrhea, rabies, febrile illness, intestinal worms, gastric diseases, lung infection, blood pressure, jaundice, vomiting, and typhoid. Correspondingly, [5, 10, 13, 52] confirmed the efficacy of this species used against intestinal parasites, abdominal pain, malaria, gastritis, fibril illness, and diarrhea in their ethnobotanical investigations in different parts of the country. Likewise, [59] confirmed its great medicinal role against malaria, convulsions, and stomachache in Uganda.

Besides, several ethno-veterinary studies conducted elsewhere in the country identified numerous therapeutic plants against different livestock ailments, demonstrating the significance of the traditional medicinal plants recorded in this study. Asfaw et al. [69], Alemneh [70], Tekle [71], Lulekal et al. [72], Eshetu et al. [73], and Yigezu et al. [74] compiled 25, 16, 20, 24, 20, and 27 medicinal plant species, respectively, in their ethno-veterinary investigations in different parts of the country. For instance, Asfaw et al. [69], Alemneh [70], Tekle [71], Lulekal et al. [72], Eshetu et al. [73], and Yigezu et al. [74] mentioned the effectiveness of *Croton macrostachyus* Hochst. ex Delile against foot rot, gastrointestinal disorders, abdominal pains, dysentery, wounds, scabies, dermatophilosis, blackleg, and trypanosomiasis. The authors also reported the medicinal use of *Gymnanthemum amygdalinum* (Delile) Sch.Bip. against jaundice, gastrointestinal disorders, abdominal pain, retained placenta, diarrhea, skin infection, and blackleg in their studied. Asfaw et al. [69], Alemneh [70], and Yigezu et al. [74] explained the therapeutic potentials of *Justicia schimperiana* (Hochst. ex Nees) T.Anderson against jaundice, swellings, gastrointestinal diseases, diarrhea, and blackleg. Asfaw et al. [69], Lulekal et al. [72], Eshetu et al. [73], and Yigezu et al. [74] described the relevance of *Allium sativum* L. against blackleg, mastitis, diarrhea, and internal body parasites.

In general, comparison of our findings with other researchers' work conducted elsewhere in the country and abroad revealed that the documented medicinal plants have wide and novel uses in the study areas and demonstrated that people from different areas employ the same medicinal plants to treat the same or different types of human ailments. This revealed that the reported medicinal plants have therapeutic and pioneering uses in the research areas and beyond. This makes it easier for further efficacy evaluation and drug synthesis from the documented plants. Their pharmacological activity should be further confirmed for use at the local and worldwide levels. Most (71%) of the identified medicinal plants were harvested from the wild (Table 3). Conservation efforts specifically targeted at medicinal plants are still a challenge in the study areas and elsewhere in the country [8, 9, 25, 47]. The well-known natural forest of the Wondo Genet areas, Munessa-Shashemene natural and plantation forest, Adaba-Dodola forest, Bale Mountains National Park, wide agro-forestry practices, and local markets are potential sources for medicinal plants. We have also seen local farmers practicing their indigenous knowledge to protect some important medicinal plants in their home gardens (Table 3). Moreover, healers discussed the challenges of cultivating plant species outside their natural habitats, as well as the need to travel considerable distances for several hours to get the necessary therapeutic plants outside their villages.

### The association between ethnobotanical knowledge and socio-demographic variables

**Fig. 4:**
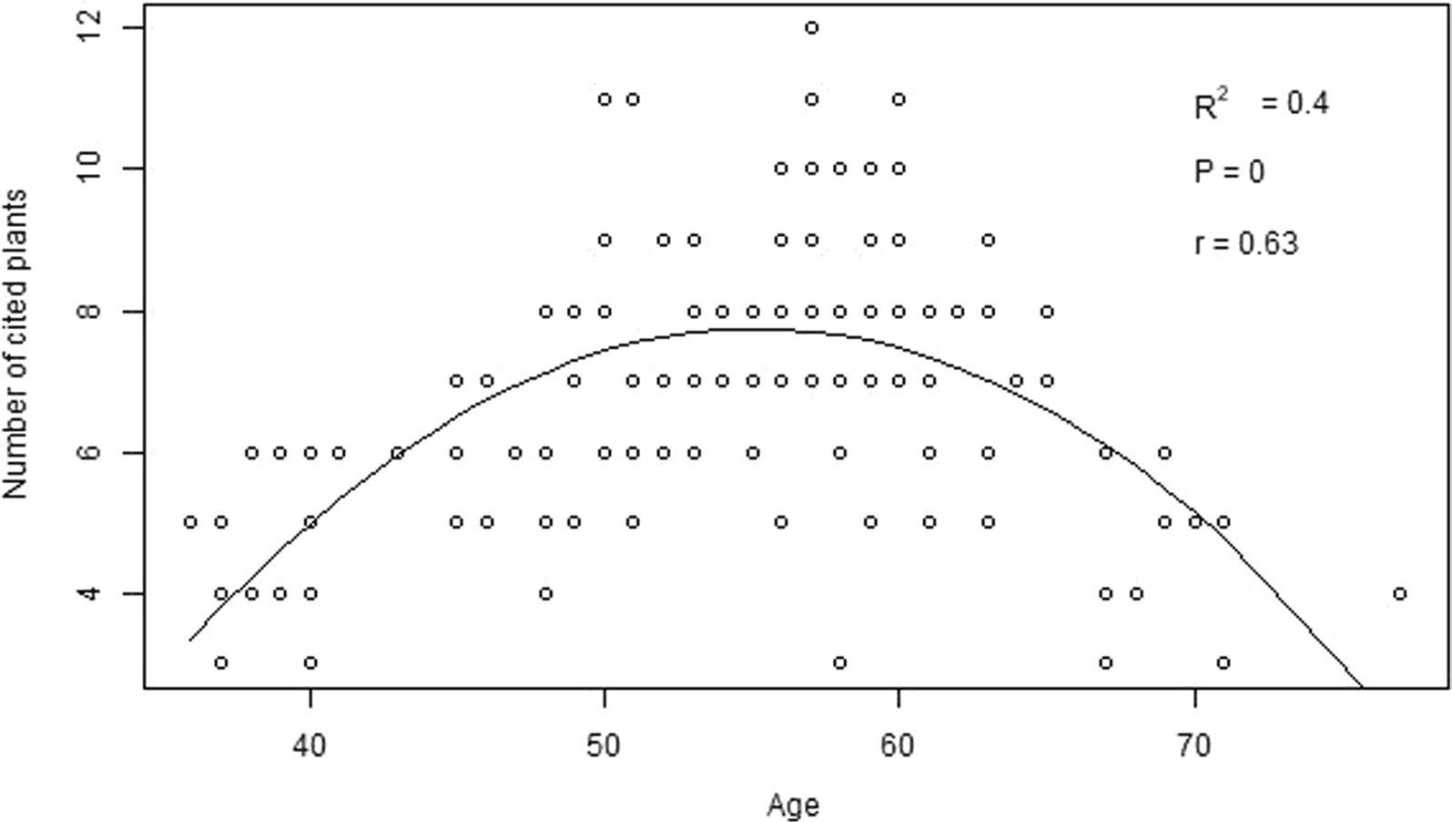
Correlation between male respondent’s knowledge of medicinal plant citation and age

**Fig. 5:**
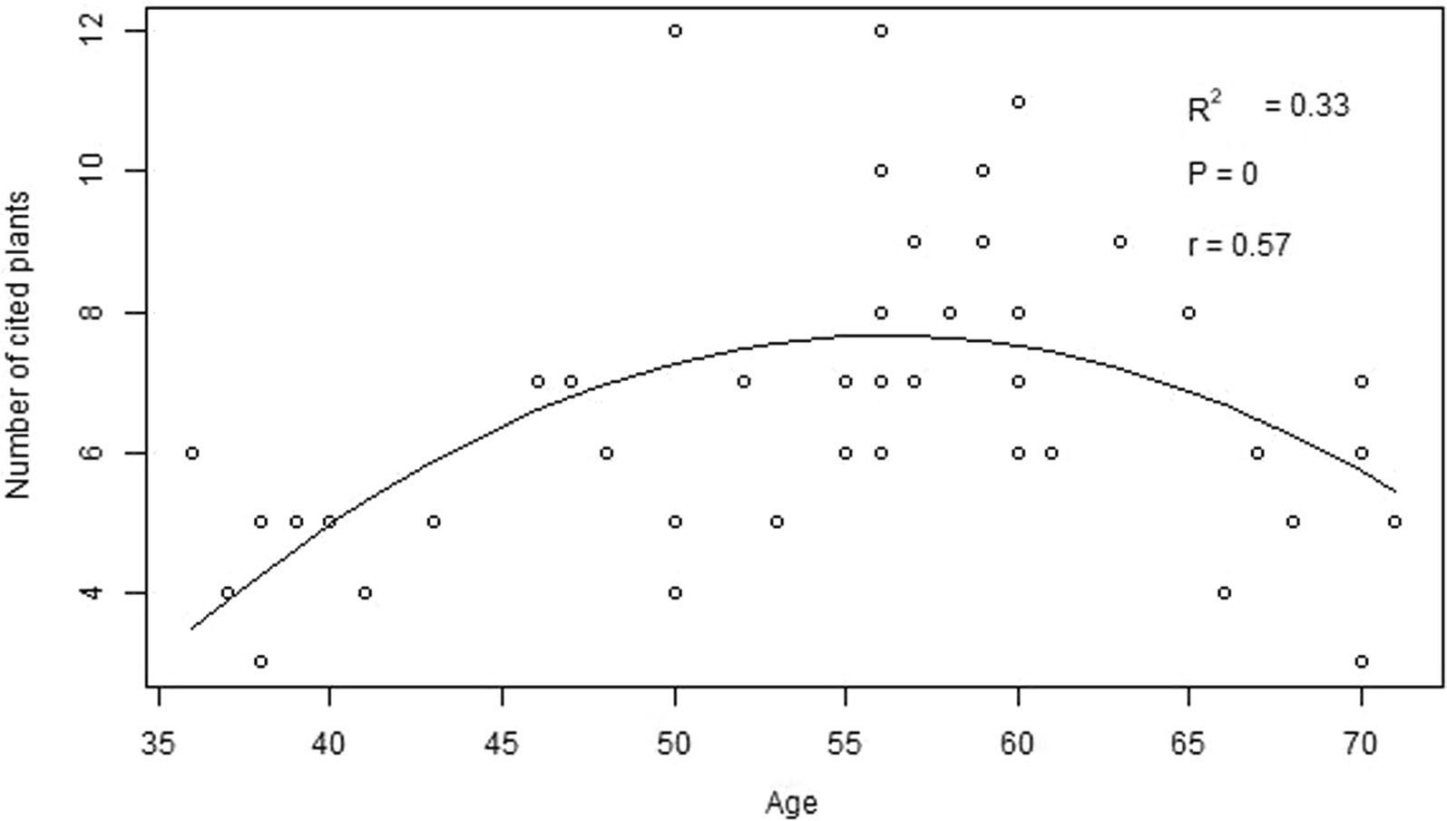
Correlation between female respondent’s knowledge of medicinal plant citation and age

The indigenous and local knowledge of medicinal plant uses of the three ethnic groups was not evenly distributed among respondents’ age groups (Table 4 and Fig. 2A). The indigenous and local knowledge of medicinal plant use is still higher among the older (age groups > 44) than among the younger (< 45 years old) across ethnic groups. Likewise, Lulekal et al*.* [9], Geta et al. [76], Eshete and Molla [10], Bekele et al. [22], Demie et al. [4], and Kidane et al. [13] reported similar findings in different parts of the country that older informants have better ethnobotanical knowledge as compared to younger. Besides, similar patterns of knowledge distribution were also witnessed abroad. Beltrán-Rodríguez et al. [77], Sharma et al. [78], Wiryono et al. [63], Amjad et al. [79], Pathy et al. [80], and Khakurel et al. [81] in Mexico, Himalaya (South Asia), Indonesia, Pakistan, DR Congo, and Nepal, respectively. Silva et al. [82] and Chekole et al. [75] also explained that older people have more opportunities for cultural interaction and familiarity with plants and their therapeutic benefits than younger. This helped them be more experienced and knowledgeable than the younger informants. The regression analysis also confirmed that the respondent's age was positively correlated with his or her ability to recognize and use ethnomedicinal plants across ethnic groups (Figs. 3, 4, 5); however, it showed a slight curvilinear relationship at the end in this study. This does not mean that the knowledge of elderly people has declined, but their openness and willingness to disclose their knowledge to outsiders are very weak, and they were too secretive and conservative in our study areas. During our discussion with them, they informed us that if all knowledge of medicinal plants is freely shared, their effectiveness in curing the illness becomes weak, particularly for those in the age group above 64. They showed disinclination to participate in the study and were reluctant to disclose their knowledge. Thus, comparatively, the individuals (age ranges between 45 and 64) mentioned a greater number of ethnomedicinal plant species than elders (Table 4). As a result, informants' disparities in ethnobotanical knowledge sharing, particularly between age groups 55–64 (older) and above 64 (elder), may have had an unintended impact on the outcome of the study.

Different ethnobotanical studies conducted elsewhere in the country also reported similar challenges. For instance, Mesfin et al. [20], in their ethnobotanical investigation in Amaro Woreda, southern Ethiopia, mentioned that the majority of participants were hesitant to disclose their knowledge of the medicinal value of the plants. They fear that their societal recognition and reputation, which they have earned due to their knowledge, will be lost, and hence they want to keep them secret. The traditional knowledge acquired from their ancestors is freely transferred within the family, preferably to the eldest son. Transfer of this knowledge to the outside world was deemed acceptable only based on substantial payment. In other studies in southern and central parts of the country, Eshete and Molla [10] and Woldeamanuel et al. [56] mentioned that most traditional healers consider traditional knowledge to be ancestral and divine, and thus, they are reluctant to disclose it to the outside world and keep it extremely secret because they think that the medicine would become ineffective if it were disseminated to others. Agize et al. [21], in their ethnobotanical investigation in southwestern Ethiopia, explained that aged informants were reluctant to disclose their ethnobotanical knowledge early as compared to other age groups, and kept it up to their last life span. Overall, this study revealed a decline in indigenous and local ethnobotanical knowledge among the younger generation across the studied ethnic groups and called for an effort to repair the observed generation gap via ongoing professional support and training of local communities to maintain traditional knowledge and practices through systematic recording. Lack of interest in traditional knowledge among young generations coupled with poor knowledge-sharing mechanisms (by word of mouth, secrecy, and only among family members) and weak policy support from concerned bodies are reported challenges for the rapid loss of indigenous and local ethnobotanical knowledge and a threat to the future potential of the country [9, 10, 59, 66, 67]. Besides, informants claimed that in recent decades, young healers had traveled to urban areas in other conditions in pursuit of work because of a lack of support from the government and a low income from traditional medication. This is important since such activities hurt the sustainability of local ethnomedicinal knowledge across generations.

Gender is another test to determine the distribution of ethnobotanical knowledge across ethnic groups. However, the difference was not statistically significant (Table 4 and Fig. 2B). Similar results were reported by [9, 10, 12–14, 66] elsewhere in the country and abroad in Nepal and Tunisia, where gender did not influence ethnomedicine claims. Thus, it was indicated that both men and women are knowledgeable about the use of traditional plant remedies, despite the relative dominance of medicinal plant traditions by men in the country, which could relate to the flow of information along the male line in the country [9, 14, 67]. Earlier studies conducted elsewhere in Ethiopia and Ecuador found that traditional medicine practitioners had nearly comparable ethnobotanical knowledge practices between the gender groups [9, 45]. In contrast, [4, 8, 59, 65, 69] elsewhere in the country and abroad in Mexico reported that significant differences were found in ethnobotanical knowledge between male and female practitioners.

Even though illiterate and lower-grade informants of the studied peri-urban districts reported more medicinal plants than higher-grade informants (Table 4 and Fig. 2C), the difference was not statistically significant. This finding revealed that all interviewed informants were knowledgeable about using traditional plant remedies regardless of their education levels, showing that education is not a factor in influencing their local knowledge of plant utilization. A similar result was reported by [12, 59] in Mexico and Nepal. In contrast, [8–10, 13, 66, 69, 70] mentioned education has effect on the health-seeking behavior of the local communities in different parts of the country and abroad in Tunisia. Our findings also showed that religion has no effect on the health-seeking behavior of the local communities; despite the fact that the majority of the people in the research areas are Protestants and Muslim religion followers, they still preferred traditional plant medicines (Table 4 and Fig. 2D). This implies that most spiritual beliefs encourage the use of therapeutic plants in the study areas. Thus, in this community, we realized that religion is not a limiting factor that affects the distribution of the local ethnobotanical knowledge system. Correspondingly, other studies conducted in the southeastern parts of Ethiopia [4, 71] also indicated that informants believed religion encouraged the use of traditional plant medicine. Contrary to this, a study by [85] revealed that religion is a limiting factor influencing the distribution of medicinal plant knowledge in India.

### Useful plants and their use values

The widespread use of plants for health treatment demonstrates an essential element of the culture [15] and is used to determine the relative significance of medicinal plants in the local communities [49]. Accordingly, *Croton macrostachyus* Hochst. ex Delile, *Zingiber officinale* Roscoe, *Albizia gummifera* C.A.Sm., and *Aloe macrocarpa* Tod. had scored the highest use value across the studied ethnic groups as a curative plant against different human ailments (Table 5). Aspects of these findings are consistent with those of other researchers [8, 10, 14, 51, 54, 55, 72, 73] who conducted ethnobotanical surveys in different parts of Ethiopia, South Africa, Bangladesh, India, and Saudi Arabia. They revealed that *Croton macrostachyus* Hochst.ex Delile and *Zingiber officinale* Roscoe had high use values against different ailments, and the in vitro investigations of [86–89] validated their efficacy on antimicrobial activities. *Albizia gummifera* C.A.Sm. has a significant use value against different human ailments in the studied communities, and the in vitro investigations of [90] confirmed the efficacy of this medicinal plant species antimicrobial activities in Cameroon. *Aloe macrocarpa* Tod. has a significant use value against different human ailments, and the in vitro investigations of [91] validated the efficacy of this medicinal plant species antimicrobial activities. *Gymnanthemum amygdalinum* (Delile) Sch.Bip., *Calpurnia aurea* (Aiton) Benth, and *Allium sativum* L. were also curative plants by all studied ethnic groups with varied use values (Table 5), and the in vitro investigations of [92–95] validated their antimicrobial activities, respectively.

In general, among the evaluated 78 important medicinal plant species, several studies conducted in Ethiopia and abroad reported different amounts of therapeutic plant use value against human ailments, which confirmed the efficacy of several traditional medicinal plants documented in this study. For instance, Chekole [14] mentioned the significant use values of 31 medicinal plant species in Gubalafto district in the northern parts of Ethiopia. *Eucalyptus globulus* Labill., *Croton macrostachyus* Hochst. ex Delile*, Achyranthes aspera* L., *Allium sativum* L., and *Solanum incanum* L. were reported medicinal plants with different use values. In another study, Agize et al. [21] reported the significant use values of *Phytolacca dodecandra* L'Hér., *Gymnanthemum amygdalinum* (Delile) Sch.Bip., *Maesa lanceolata* Forssk., and *Eucalyptus globulus* Labill. in the studied communities. Abroad, Shaheen et al. [96], Ishtiaq et al. [97], Faruque et al. [61], Gupta et al. [68], and Al-robai et al. [64] revealed 9, 7, 8, 6, and 14 significant medicinal plants in Pakistan, Bangladesh, India, and Saudi Arabia. Besides, it was also confirmed by Eshete and Molla [10] that repeatedly used plants are more likely to be biologically active and indicate the popularity of the local medicinal flora in the local culture. Therefore, these species should be prioritized for conservation. Their preferred uses may place their populations under threat due to overharvesting.

### The general understanding among informants

Consensus analysis is a critical tool for establishing a comparative evaluation of the level of informant's agreement on the use of medicinal plants [50]. Beyond that, it would provide dependability for every claim supported by reliable facts in ethnobotanical investigations [98]. As a result, the majority of the clusters generated in this study confirmed the unique and shared knowledge of each ethnic group on similar or different plant species against a variety of ailments and the diffusion of information and cultural linkage (Table 6). Most of the clusters had an informant consensus value greater than 50, indicating that they might all be evaluated for validation in support of their traditional use. These demonstrate a high level of agreement among the informants regarding the use of specific plants to treat the similar disorder. Particularly, the four use categories (Circulatory system disorders, febrile illness, reproductive organ disorders, and Bad/evil spirit) scored high ICF values across the studied ethnic groups. Different studies conducted elsewhere in the country also reported high ICF value for the same illness categories [8, 10, 13, 14, 52]. Lulekal et al. [9] explained that the high informant consensus values obtained indicate reasonably high reliability of informants on the use of traditional medicinal plants, which are thought to have better potency and contain more biologically active ingredients in disease treatment.

In this study, six medicinal plant species gained common consensus among the studied ethnic groups against febrile illnesses, glandular problems, reproductive organ illnesses, respiratory organ illnesses, and bad or evil spirit complication categories (Table 6). *Allium sativum* L. obtained a common consensus against typhoid and common cold; *Croton macrostachyus* Hochst. ex Delile against dizziness and gonorrhea; *Gymnanthemum amygdalinum* (Delile) Sch.Bip. against malaria; *Moringa stenopetala* (Baker f.) Cufod. against glad-related complications; *Ruta chalepensis* L. against gonorrhea; and *Withania somnifera* (L.) Dunal against bad or evil spirits. Ethnobotanical studies conducted elsewhere in the country also revealed similar kinds of findings. For instance, [5] reported the efficacy of *Allium sativum* L. against cold and febrile illness (general malaise), *Croton macrostachyus* Hochst. ex Delile against febrile illness (general malaise), *Gymnanthemum amygdalinum* (Delile) Sch.Bip. against malaria, and *Withania somnifera* (L.) Dunal against bad or evil spirits. In another study, [14] revealed the significance of *Croton macrostachyus* Hochst. ex Delile against gonorrhea and febrile illnesses and *Withania somnifera* (L.) Dunal against bad or evil spirit complications. In the southern parts of the country, Tefera and Kim [8] also reported the medicinal value of *Ruta chalepensis* L. against gonorrhea. Again, in southern and southwestern parts of Ethiopia, [42, 52] confirmed the efficacy of *Allium sativum* L. against common cold. Similarly, [10, 52] reported the ethnomedicinal effectiveness of *Croton macrostachyus* Hochst. ex Delile against gonorrhea. Also [10, 42] reported the significance of *Withania somnifera* (L.) Dunal against bad or evil spirits in the southern parts of the country. However, *Moringa stenopetala* (Baker f.) Cufod. was found to be a novel finding in the study areas against gland-related problems. Therefore, further consideration and studies are needed to evaluate the information about the phytochemical and pharmacological potentials of the recorded ethnomedicinal plants for wider utilization.

Contrary to this, different consensuses were also noted among the three ethnic groups on some medicinal plants against circulatory system illness categories (snake venom). Those are *Gymnanthemum auriculiferum* (Hiern) Isawumi, which got the highest consensus within the Gedeo ethnic group against snake venom, whereas *Solanum incanum* L. and *Searsia natalensis* (Bernh. ex Krauss) F.A.Barkley were within the Oromo and Sidama ethnic groups, respectively, (Table 6). Besides, as compared to the Oromo ethnic group, the Sidama ethnic group showed great consensus to treat mental (rabies) disorders using *Antiaris toxicaria* (J.F.Gmel.) Lesch., and *Justicia schimperiana* (Hochst. ex Nees) T.Anderson. The Gedeo ethnic group informant has shown a unique agreement on the abruptness lightning complications using *Croton macrostachyus* Hochst. ex Delile *and Ensete ventricosum* (Welw.) Cheesman and against musculoskeletal illness using *Calpurnia aurea* (Aiton) Benth. and *Croton macrostachyus* Hochst. ex Delile. Informant consensus values near or zero indicate low informant agreement, which could be attributed to the community's use of different species for the same ailments [36, 45] (Table 6). Several studies conducted elsewhere also confirmed that conditions such as circulatory system disorders, gastrointestinal diseases, respiratory system disorders, Evil/bad spirits, and febrile illness were disease categories recognized as being efficiently treated by traditional plant medicine [8, 9, 39, 58, 60, 61].

### Species consensus of informants

The fidelity level (FLs), relative popularity level (RPL), and rank-order priority (ROP) values are considered to determine for which illness a particular plant species is more effective in the study areas. In general, a rank-order priority (ROP) of 100% for a specific plant species indicates that all of the use reports mentioned were the same and an excellent choice for treating particular ailments [5, 62, 63]. In contrast, the low fidelity levels and low rank-order priority indicate the plant species will be employed for diverse purposes, according to [59]. The reported highest fidelity level values for *Lactuca inermis* Forssk., *Moringa stenopetala* (Baker f.) Cufod., *Withania somnifera* (L.) Dunal., *Allium sativum* L., *Citrus limon* (L.) Osbeck, *Ricinus communis* L., Schinus *molle* L., *Antiaris toxicaria* (J.F.Gmel.) Lesch., *Brucea antidysenterica* J.F.Mill., *Echinops kebericho* Mesfin, *Ocimum jamesii* Sebald, *Afrocarpus falcatus* (Thunb.) C.N.Page and *Searsia natalensis* (Bernh. ex Krauss) F.A.Barkley could be considered as evidence of the high healing potential of these plants against the corresponding diseases in the study areas (Table 7). In contrast, *Ricinus communis* L., and *Allium sativum* L. scored the lowest points against tonsillitis and fever, respectively. The revealed results confirmed that ethnic groups have diverse ethnobotanical knowledge of specific plants and disease conditions.

Different ethnobotanical investigations conducted elsewhere in the country and abroad revealed considerable evidence about the therapeutic potentials of some of the selected medicinal plants in the study areas. Consistently, Tefera and Kim [8] revealed the medicinal potential of *Moringa stenopetala* (Baker f.) Cufod. against cardiovascular diseases and [5, 10, 14] reported the efficacy of *Withania somnifera* (L.) Dunal. against bad or evil spirits in different parts of the country. Similarly, another study [5] confirmed the effectiveness of *Allium sativum* L. against common cold in the south-central parts of the country. In addition to our findings, [5, 9] reported the ethnomedicinal effectiveness of *Echinops kebericho* Mesfin against evil spirits, general malaise, abdominal pain, diarrhea, and amoebiasis in the central and south-central parts of the country. In other ethnobotanical investigations, [5, 9, 84] again elucidated the ethnomedicinal role of *Allium sativum* L. against skin diseases, malaria, toothache, general malaise, and tuberculosis. In Ethiopia and abroad, [5, 13, 14, 85] revealed the ethnomedicinal effectiveness of *Withania somnifera* (L.) Dunal. against febrile illnesses, general malaise, liver disorders, kidney pain, and blood purification. Thus, the use of the same plant species for related or dissimilar ethnomedicinal applications in different countries is a reliable indication of the recorded plant species' bioactivity potential, and the presence of a certain ailment in the area and the widespread use of traditional plant medicine [9, 64]. The findings will be used in future research to confirm the bioactivity of certain medicinal plants employed by traditional healers and boost their acceptance in broader healthcare systems in the country and abroad.

### Use diversity and cultural roles of some medicinal plants

The medicinal plant species documented in this study are also cited for multiple local uses besides their medicinal role (Table 3). All medicinal plant species, particularly trees, shrubs, and herbs, were believed to be beneficial to the environment. Maintaining ecosystems through erosion regulation, soil enhancement, fodder and shelter for wild animals, and climate regulation were among the major roles. The mentioned environmental services were justified in the sense that each plant species has a role in supporting balanced biophysical systems. Based on the informants in our study, of the 189 identified medicinal plant species, 30 were reported for additional uses as food and 40 as livestock fodder, indicating their supplemental role in supporting the livestock and livelihood wealth of the study areas (Table 3). Additionally, more than 100 medical plant species were utilized as fuel (charcoal and firewood), whereas 16 were noted for timber production, which is the primary source of income for the majority of local communities. About 52 medicinal plant species were also employed for local construction uses, including 22 for social services such as cultural gathering under shade of those trees to resolve local conflict (Table 3). Around ten medicinal plant species were also identified as spices to food in the study areas. These findings demonstrated that the breadth of indigenous and local knowledge practices among local people in using plant resources for different purposes. On the other hand, the medicinal use values of plant species employed across different use categories demonstrated the relative importance of various plant species in people's everyday lives. Different studies conducted elsewhere in the countries also explained the diverse potentials of the documented medicinal plant species beyond their medicinal roles. For instance, Gurebiyaw et al. [102] mentioned the ecological significance of *Albizia gummifera* (J.F.Gmel.) C.A.Sm., *Cordia africana* Lam., and *Croton macrostachyus* Hochst. ex Delile on soil fertility enhancement. Negash [103] explained the promising options of *Afrocarpus falcatus* (Thunb.) C.N.Page, *Cordia africana* Lam., *Croton macrostachyus* Hochst. ex Delile, *Ekebergia capensis* Sparrm., *Millettia ferruginea* (Hochst.) Hochst. ex Baker, and *Vachellia seyal* (Delile) P.J.H.Hurter in maintaining soil fertility, soil regeneration and water conservation, provision of shade or shelter, as well as for windbreak.

In the study areas, it was also noted that farmers valued very much certain medicinal plant species for traditional farming tools and local buildings. *Afrocarpus falcatus* (Thunb.) C.N.Page and *Olea europaea subsp. cuspidata* (Wall. & G.Don) Cif. were preferred plant species for making three traditional Ethiopian farming tools, “Wanjoo, Gindii and Maneqoo” in the Oromo ethnic group and “Mofaara, Qanbaraa, and Maneqoo” in the Gedeo and Sidama ethnic groups, which are commonly drawn by a pair of oxen. Additionally, the wood and leaves of *Olea europaea subsp. cuspidata* (Wall. & G.Don) Cif. are burned to produce a characteristic smoke, which serves as a good flavor for fermenting milk and making local alcohol (e.g., locally known as *Tella* and *Tej*). Another intriguing aspect is that the Sidama ethnic group tendency for traditional house construction using bamboo species and *Juniperus procera* Hochst.Ex Endl. throughout the generations. The Sidama ethnic group is located in the southern Ethiopia and has unique expertise in how to create a stunning beehive-shaped traditional house entirely built of homogeneous woven bamboo. *Juniperus procera* Hochst. ex Endl. tree poles are used in the foundation because local communities think this species has natural resistance to microbes and termites and is long-lived. Dainty strands of bamboo are set around the edge of the house. The walls are made by parting the bamboo into more modest strips. The interior walls include two designs locally known as "*Hilo* and *Himbiro*".

### Public health and roles of medicinal plants

Traditional medicine and medicinal plants have continued to play an essential role in the public healthcare system, both in the study areas and in elsewhere in the country. Because it is understandable that, in Ethiopia, almost 80% of the population uses traditional medicine, which is primarily based on medicinal plants [104], and more than 95% of folk medicine decoctions are derived from medicinal plants [105]. According to Lulekal et al. [9], the documented medicinal plants with high informant consensus (IC) and rank-order priority (ROP) values have promising potential against different pathogens and play a great role in maintaining the general public health of local communities and beyond. For instance, the authors reported high informant consensus and rank-order priority values for *Afrocarpus falcatus* (Thunb.) C.N.Page, *Allium sativum* L., *Croton macrostachyus* Hochst. ex Delile, *Echinops kebericho* Mesfin, *Gymnanthemum amygdalinum* (Delile) Sch.Bip., *Moringa stenopetala* (Baker f.) Cufod., *Ruta chalepensis* L., *Withania somnifera* (L.) Dunal, *Ricinus communis* L., and *Zingiber officinale* Roscoe (Tables 6 and 7) are indications for their role in maintaining the community’s health against different communicable and non-communicable diseases in the study areas. Besides, in vitro, investigations conducted by [79, 83, 85, 97–101] confirmed their effectiveness against many disease-causing pathogens in the country and abroad.

## Conclusion

The medicinal plant resources in the studied rural–urban interface areas are considerably high. The associated knowledge of the local people is deep-rooted in the time-honored practices of traditional plant medicine, which depended heavily on medicinal plant species to cure different ailments. The study revealed a generational gap across ethnic groups in medicinal plant knowledge acquisition. Thus, older generations have continued to play a role as reservoirs for indigenous and local ethnobotanical knowledge practices. The use values of the most important medicinal plant species were evaluated and revealed considerable variability among the ethnic groups studied. *Croton macrostachyus* Hochst. ex Delile, *Albizia gummifera* (J.F.Gmel.) C.A.Sm., *Zingiber officinale* Roscoe*, Aloe macrocarpa* Tod., *Gymnanthemum amygdalinum* (Delile) Sch.Bip., *Ruta chalepensis* L., and *Calpurnia aurea* (Aiton) Benth. were noted species with high use value. Knowledge dissemination among informants was highest in the categories of circulatory system disorders, febrile illnesses, and reproductive organ complications across ethnic groups. The curative potential of some medicinal plants was evaluated and revealed significant fidelity values across ethnic groups. *Lactuca inermis* Forssk., *Moringa stenopetala* (Baker f.) Cufod., *Withania somnifera* (L.), *Allium sativum* L., *Citrus limon* (L.) Osbeck, *Ricinus communis* L., *Schinus molle* L., *Antiaris toxicaria* (J.F.Gmel.) Lesch., *Brucea antidysenterica* J.F.Mill., *Echinops kebericho* Mesfin, *Ocimum jamesii* Sebald, *Afrocarpus falcatus* (Thunb.) C.N.Page, and *Searsia natalensis* (Bernh. ex Krauss) F.A.Barkley are among the top ones. Thus, the revealed results will provide relevant information for further research focusing on investigating the bioactive compounds of some selected curative plants, sustaining indigenous and local ethnobotanical knowledge, and the associated most important medicinal flora. Besides, providing professional support to manage the ongoing dynamics and maintain the vast erosion of indigenous and local knowledge is vital.

## Data Availability

All data generated or analyzed in this study are included in this manuscript.

## Abbreviations

CSA: Central Statistical Agency of Ethiopia
US: Use value
ICF: Informant consensus factor
FL: Fidelity level
RPL: Relative popularity level
ROP: Rank-order priority
SD: Standard deviation
FC: Frequency of citation
UR: Use reports
NU: Number of uses
